# Genotypic and phenotypic profiling of 127 *Legionella pneumophila* strains: Insights into regional spread

**DOI:** 10.1371/journal.pone.0307646

**Published:** 2024-07-19

**Authors:** Andrea Colautti, Marcello Civilini, Renzo Bortolomeazzi, Marinella Franchi, Antonella Felice, Stefano De Martin, Lucilla Iacumin

**Affiliations:** 1 Department of Agricultural, Food, Environmental and Animal Science (Di4A), University of Udine, Udine, Italy; 2 Laboratory of Microbiology, ARPA–Regional Agency for Environmental Protection Friuli Venezia Giulia, Udine, Italy; Maria Curie-Sklodowska University, POLAND

## Abstract

Given the recent global surge in Legionnaires’ disease cases, the monitoring of *Legionella pneumophila* becomes increasingly crucial. Epidemiological cases often stem from local outbreaks rather than widespread dissemination, emphasizing the need to study the characteristics of this pathogen at a local level. This study focuses on isolates of *L*. *pneumophila* in the Italian region of Friuli Venezia Giulia to assess specific genotype and phenotype distribution over time and space. To this end, a total of 127 *L*. *pneumophila* strains isolated between 2005 and 2017 within national surveillance programs were analysed. Rep-PCR, RAPD, and Sau-PCR were used for genotypic characterization, while phenotypic characterization was conducted through fatty acids analysis. RAPD and Sau-PCR effectively assessed genetic characteristics, identifying different profiles for the isolates and excluding the presence of clones. Although Sau-PCR is rarely used to analyse this pathogen, it emerged as the most discriminatory technique. Phenotypically, hierarchical cluster analysis categorized strains into three groups based on varying membrane fatty acid percentages. However, both phenotypic and genotypic analyses revealed a ubiquitous profile distribution at a regional level. These results suggest an absence of correlations between strain profiles, geographical location, and isolation time, indicating instead high variability and strain dissemination within this region.

## 1 Introduction

In recent years, there has been a steady increase in the incidence of legionellosis cases, a water-borne disease caused by exposure to contaminated water, with *Legionella pneumophila* (*Lp*) [1] being the main etiological agent, observed both in Europe [2] and the United States [3]. Various factors may correlate with this surge, including climatic fluctuations such as elevated temperatures and changes in precipitation patterns, which have been reported by several authors as impacting the proliferation of *Lp* in aquatic environments. These conditions increase the risk of human exposure and, consequently, a rise in legionellosis cases [4–9]. Furthermore, poor water management and changes in distribution systems can enhance *Lp* growth in water facilities, raising the risk of human exposure. This was exemplified during the COVID-19 pandemic, wherein the discontinuation of several water facilities’ utilization fostered water stagnation [10]. Additionally, the pandemic has increased the number of individuals potentially at risk, as recovered patients often have compromised respiratory systems. The aging population is an additional contributory factor to the increased susceptibility to this infection, as elderly or immunocompromised individuals are at a higher risk of developing severe forms of the disease [11]. Moreover, international travel and globalization can facilitate the spread of local outbreaks of legionellosis through the movement of infected individuals, with numerous cases reported among travellers who have stayed in accommodation facilities such as hotels or cruise ships [12]. It should also be considered that statistics on the incidence of legionellosis may be influenced by advancements in diagnostic techniques and increased awareness among medical personnel, leading to a more accurate detection of legionellosis cases. This may seemingly indicate an increase in cases when, in reality, the disease is simply being diagnosed and identified more effectively [13].

Regarding its dissemination, it is essential to note that legionellosis is typically associated with localized outbreaks [14] rather than a generalized increase on a global scale [3]. Episodes of legionellosis are frequently linked to specific sources of exposure in particular locations or structures, as environmental conditions and risk factors can vary significantly among different places [15]. Therefore, local monitoring enables the improvement of prevention and control strategies tailored to the specific conditions of each area, facilitating timely outbreak detection. The swift identification and response to local outbreaks contribute to safeguarding public health, reducing the risk of disease spread, and limiting the number of cases. For these reasons, it is also important to characterize the isolates at both genotypic and phenotypic level, to identify their characteristics and more effectively counteract their presence at both environmental and medical level in the case of infected patients.

In this regard, besides Pulsed Field Gel Electrophoresis (PFGE), other techniques such as RAPD, rep-PCR, and Sau-PCR have proven effective in *Lp* genotypic characterization. These methodologies, previously employed in other contexts to differentiate between various *Legionella* spp., were deemed advantageous for their rapidity and cost-effectiveness [16]. In addition to species differentiation, some studies have highlighted their effectiveness in discriminating among similar strains of *Lp*, even distinguishing their serotype [17–19]. Regarding phenotypic characterization, one of the most effective methods reported is the typing of membrane fatty acids. Initial studies on the cellular fatty acid profile of *Lp* were conducted on pathogenic strains isolated from patients involved in the 1976 pneumonia outbreak [20]. The primary phospholipid constituting the membrane of *Lp* is phosphatidylcholine (PC), a phospholipid typical of the cellular membranes of eukaryotic organisms. The presence of PC in the bacterial membrane can influence the interaction with host cells, as this phospholipid is commonly associated with the cellular membranes of host organisms. Furthermore, *Lp* is recognized for its ability to modify the lipidic composition of its membrane in response to the surrounding environment, including the biological fluids of the host [21]. These alterations can modulate the interaction with host cells. Collectively, the unique lipid composition of *Lp* membrane influences how the bacterium interacts with host cells. This interaction is crucial for *Lp* ability to adapt to the hostile host environment and evade or circumvent the host immune response, thereby contributing to its capacity to cause Legionnaires’ disease [22]. Furthermore, the phenotypic plasticity of the *Lp* membrane, modulated by fatty acids, and its ability to utilize exogenous choline allow it to adapt to environmental variations, including the intracellular environment of amoebae in which it thrives. Additionally, the atypical structure of lipid A contributes to its immunomodulatory properties. The lipid A structure of *Lp* often features a long and complex side chain, which can vary depending on the serotype. This atypical structure is considered one of the reasons why *Legionella* spp. can partially evade the host immune response, modulate the inflammatory response, and protect the bacterium from esterases within amoebae [23].

In this context, this study aimed to deepen the understanding of the spread of *Lp* in the Italian region of Friuli Venezia Giulia in northeastern Italy. To this end, the study investigated the presence of correlations between the spatio-temporal distribution of various genotypic and phenotypic profiles of 127 *L*. *pneumophila* strains. These strains were isolated from tap water in different types of buildings identified as sources of legionellosis outbreaks between 2005 and 2017. The investigation covered various municipalities and was part of the national surveillance plans conducted by the Regional Environmental Protection Agency of Friuli Venezia Giulia (ARPA FVG). For the analysis, two approaches were used, focusing on conventional and well-established techniques for studying this pathogen. The first characterization method concentrated on the genetic aspect, assessing the differences between the strains using RAPD-PCR, rep-PCR, and Sau-PCR techniques. The second approach involved the phenotypic typing of membrane fatty acids in different strains. The results obtained from these methods provided an initial characterization and overview of the differentiation of *L*. *pneumophila* strains isolated in this region, thus laying the foundation for more in-depth studies using Whole Genome Sequencing (WGS) techniques.

## 2 Materials and methods

### 2.1 Regional strains isolation

The strains examined in this study were collected as part of national surveillance plans in the Friuli Venezia Giulia, a northeastern region of Italy, between 2005 and 2017. This analysis, conducted by the Regional Agency for Environmental Protection of Friuli Venezia Giulia (ARPA FVG), aimed at identifying the sources of legionellosis outbreaks by sampling tap water systems of buildings presumed to be sources of infection. Samples were obtained following the ISO 11731:2017 protocol. Briefly, 1-Liter water samples were membrane filtered (Pall Corporation, USA). The filtering membrane was re-suspended in a sterile tube with 10 mL of water from the same sample to resuspend the microorganisms. Then, 0.1 mL of the final sample was inoculated on Legionella Agar (Biolife, Italy) growth media. Colonies considered positive underwent presumptive identification through sub-culturing each colony on both Legionella Agar plates (Biolife, Italy) and Legionella Agar without Cysteine plates (Biolife, Italy). Inoculated plates were incubated in a jar under microaerophilic conditions using CampyGen™ 2.5 L in a thermostat at 36°C for up to 10 days. Colonies grown exclusively on the former were considered as *Legionella* spp.

Species and serogroup identification were conducted based on antigenic reactions using latex agglutination serological tests with monoclonal antibodies (Legionella rapid latex test kit, Mascia Brunelli S.p.a., Italy) and real-time PCR following Annex 6 of the Italian national Guidelines [24]. If the definitive result was positive, *L*. *pneumophila* isolates were preserved at -80°C in Cryobanks (Mast House, UK) until further analyses were performed.

### 2.2 Reference strains

For the comparison of the tested strains, four strains of different *Legionella pneumophila* serogroups and other strains of *Legionella* spp. were employed. The following strains were purchased in lyophilized form from the Leibniz-Institut DSMZ-Deutsche Sammlung von Mikroorganismen und Zellkulturen GmbH (DSMZ) collection: *Legionella pneumophila* DSM 7513 serogroup 1 (STD1), *Legionella pneumophila* DSM 25071 serogroup 2 (STD2), *Legionella pneumophila* DSM 25182 serogroup 6 (STD6), *Legionella pneumophila* DSM 25184 serogroup 8 (STD8), *Legionella longbeachae* DSM 10572, *Legionella bozemanii* DSM 16523, *Legionella micdadei* DSM 16640, *Legionella dumoffii* DSM 17625, and *Legionella gormanii* DSM 25296.

### 2.3 DNA extraction

For all strains, DNA extraction was performed using the modified Querol method [25]. Cryopreserved colonies, cultured for 5 days on Legionella Agar, were collected using a sterile 10 μL loop and transferred to a sterile tube containing 500 μL of Solution A (50 mg/mL Lysozyme, 1 M sorbitol, 0.1 M EDTA, in MilliQ water, final pH = 7.4) and incubated at 37°C for 2 h. Subsequently, the tubes were centrifuged at 8000 × g for 10 minutes, the supernatant was discarded, and the obtained pellet was resuspended in 500 μL of Solution B (50 mM Tris-HCl, 20 mM EDTA, in MilliQ water, final pH = 7.4). Samples were then incubated in a water bath at 65°C for 30 minutes, with the addition of 50 μL of 10% SDS Solution. After the incubation period, 200 μL of 5% KAc solution were added, and the samples were kept on ice for 30 minutes. Subsequently, the tubes were centrifuged at 14000 × g for 5 minutes. The supernatant, containing DNA, was transferred to new sterile tubes and 1 mL of absolute ethanol (Carlo Erba, Milan, Italy) was added. The tubes were then centrifuged at 14000 × g for 10 minutes, the supernatant was discarded, and 500 μL of cold 70% ethanol were further added. The tubes were centrifuged again at 14000 × g for 5 minutes. After removing the supernatant ethanol, the DNA pellet was air-dried overnight at 37°C. The pellet was then resuspended in 50 μL of sterile MilliQ water, and 1 μL of RNase (Sigma-Aldrich, USA) was added for a 1-hour incubation at 37°C to digest co-extracted RNA. DNA quantification was performed using Nanodrop One (Thermo Scientific, USA) and standardized to 50 ng/μL for the following analyses.

### 2.4 Rep-PCR, RAPD, and Sau-PCR analyses

The molecular characterization was performed according to Iacumin et al. 2020 [26]. Rep-PCR analysis was performed using (GTG)_5_ primer (5’-GTGGTGGTGGTGGTG-3’) [27], using the following reaction mix: 10 mM Tris-HCl (pH 8.3), 50 mM KCl, 1.5 mM MgCl_2_, 0.2 mM each dNTP, 1 μM primer (GTG)_5_, 1.25 U Taq-polymerase (Applied Biosystems, USA), and 100 ng of DNA, for a total volume of 25 μL. The reactions were carried out using a Euroclone Thermal Cycler (Celbio, Milan, Italy) and the amplification protocol consisted of 31 cycles of denaturation at 94°C for 3 s followed by one step at 92°C for 30 s, annealing at 40°C for 1 min and extension at 65° C for 8 min. The initial denaturation was at 95° C for 2 min and the final extension at 65° C for 8 min.

RAPD analysis was performed using the M13 primer (5’-GAG GGT GGC GGT TCT-3’) [28], using the following reaction mix: 10 mM Tris-HCl (pH 8.3), 50 mM KCl, 1.5 mM MgCl_2_, 0.2 mM each dNTP, 1 μM primer M13, 1.25 U Taq-polymerase (Applied Biosystems, USA) and 100 ng of the extracted DNA for a total volume of 25 μL. The reactions were carried out in a Euroclone Thermal Cycler (Celbio, Italy) and the amplification protocol consisted of 35 cycles of 94° C for 1 min, 38° C for 1 min, ramp to 72° C at 0.6° C/s, and 72° C for 2 min. At the beginning of the reaction, an initial denaturation step at 94° C for 5 min was performed, followed by a final extension at 72° C for 5 min.

Sau-PCR analysis was performed by digesting 200 ng of DNA overnight at 37°C with 1 μL of Sau3AI restriction endonuclease (10 U/μL) in a final volume of 20 μL. After enzymatic restriction, the amplification was performed using the SAG1 primer (5’-CCGCCGCGATCAG-3’) [29] with the following reaction mix: 10 mM Tris–HCl (pH 8.3), 50 mM KCl, 1.5 mM MgCl_2_, 0.2 mM each dNTPs, 2 μM primer SAG1, 1.25 U Taq-polymerase (Applied Biosystems, USA), and 1 μL of the digested DNA for a total volume of 50 μL. The reactions were carried out using a Euroclone Thermal Cycler (Celbio, Italy) with the following protocol: 25° C for 5 min, ramp to 60°C at 0.1°C/s, 60°C for 30 s, 2 cycles of 95°C for 1 min, 50°C for 15 s, ramp to 25°C at 0.1°C/s, ramp to 50°C at 0.1°C/s, 50°C for 30 s, 35 cycles of 94°C for 15 s, 46°C for 1 min, 65°C for 2 min, and a final extension at 65°C for 2 min.

PCR products obtained from these techniques were separated in a 1.5% (w/v) agarose gel in TBE 0.5X at 120 V, for 4, 6, and 3 h for RAPD, rep-PCR, and Sau-PCR, respectively. Staining was performed for 30 min at the end of the electrophoretic run in TBE 0.5X buffer containing ethidium bromide 0.25 μL/mL (Sigma- Aldrich, St. Louis, USA). Digital images of the gels were acquired using the BioImaging System GeneGenius imaging software (Syngene, Italy).

### 2.5 Cellular fatty acid analysis

After revitalization and further streaking of the cryopreserved strains on Legionella Agar (36°C for 5 days), strains were cultured on Legionella Agar for 96 h at 36°C for the analysis of cellular fatty acids. As reported in the literature, the fatty acid composition of *L*. *pneumophila* is influenced by both incubation time [30, 31] and growth phase [32]. To ensure consistency and avoid growth-induced variations, all strains were carefully cultivated under identical conditions and harvested after 96 hours of growth, in accordance with previous studies that investigated fatty acid composition in *Legionella* spp. [31, 32]. For the analysis, aliquots of about 80 mg (wet weight) of cells were removed from the surfaces of the agar plates and placed in test tubes equipped with Teflon-lined screw caps. Fatty acid methyl esters (FAMEs) were prepared by saponification, methylation, and extraction, following the method reported by Sasser (1990) [33]. After the final washing step, the extract containing the FAMEs was dried with anhydrous sodium sulphate, transferred to an autosampler vial and the volume reduced to about 0.5 mL under a nitrogen stream.

### 2.6 Gas chromatography-flame ionization detection (GC-FID)

A Trace 1300 gas chromatograph, equipped with a flame ionization detector, a split/splitless injector, and an autosampler AL 1310 (Thermo Fisher Scientific, Italiy), was used. The separation was performed using a fused silica capillary column HP-5MS UI, 30 m x 0.25 mm, 0.25 μm film thickness (Agilent Technologies, Italy). The initial oven temperature was set at 140°C and increased to 300°C at 5°C/min. The injector and detector temperatures were 280 and 300°C, respectively. Helium was used as the carrier gas at a flow rate of 1.0 mL/min. The injection volume was 1 μL, and the split ratio was 1:10.

### 2.7 Gas chromatography-mass spectrometry (GC–MS)

A Agilent Technologies 7890B gas chromatograph, coupled to a quadrupolar mass detector (Agilent Technologies, Italy), was used. The separation was performed using a fused silica capillary column HP-5MS UI, 30 m x 0.25 mm, 0.25 μm film thickness (Agilent Technologies Italia, Italy). The initial oven temperature was set at 140°C and increased to 280°C at 5°C/min. The injector, transfer line, source, and quadrupole temperatures were 250, 280, 175, and 150°C, respectively. Helium was used as carrier gas at a flow rate of 1.0 mL/min. The injection volume was 1 μL, and the split ratio was 1:10. The mass spectra were recorded under electron impact (EI) at 70 eV.

### 2.8 Identification of fatty acids

FAMEs identification from GC-FID chromatograms was achieved by comparing retention times and mass spectra with those of authentic standards Bacterial Acid Methyl Ester (BAME) Mix (Supelco, Italy). For the compounds not present in the BAME mix, tentative identification was obtained by comparing the linear retention index, calculated by injecting a mixture of n-alkanes C7-C30 (Supelco, Sigma-Aldrich, Milan, Italy) under the same conditions, with literature data and the mass spectra with the mass spectral library NIST 14 of the instrument. Quantitative analysis was carried out by the percentage peak area method, considering all the compounds to have the same response to the flame ionization detector.

### 2.9 Statistical analysis

The fingerprint analysis of RAPD, rep-PCR, and Sau-PCR was carried out with the Gel Compare II software Version 4.1 (Applied Maths, Sint-Martens-Latem, Belgium), calculating their similarity using Sørensen–Dice correlation coefficient. Dendrograms were obtained using the Unweighted Pair Group Method with Arithmetic Average (UPGMA) clustering algorithm [34]. All further statistical analyses were conducted using R v4.1.2 software. For the creation and plotting of the different images, the ape [35], phangorn [36], plotly [37], dendextend [38], and ggplot2 [39] libraries were used.

## 3 Results

### 3.1 Dataset description

**Fig 1:**
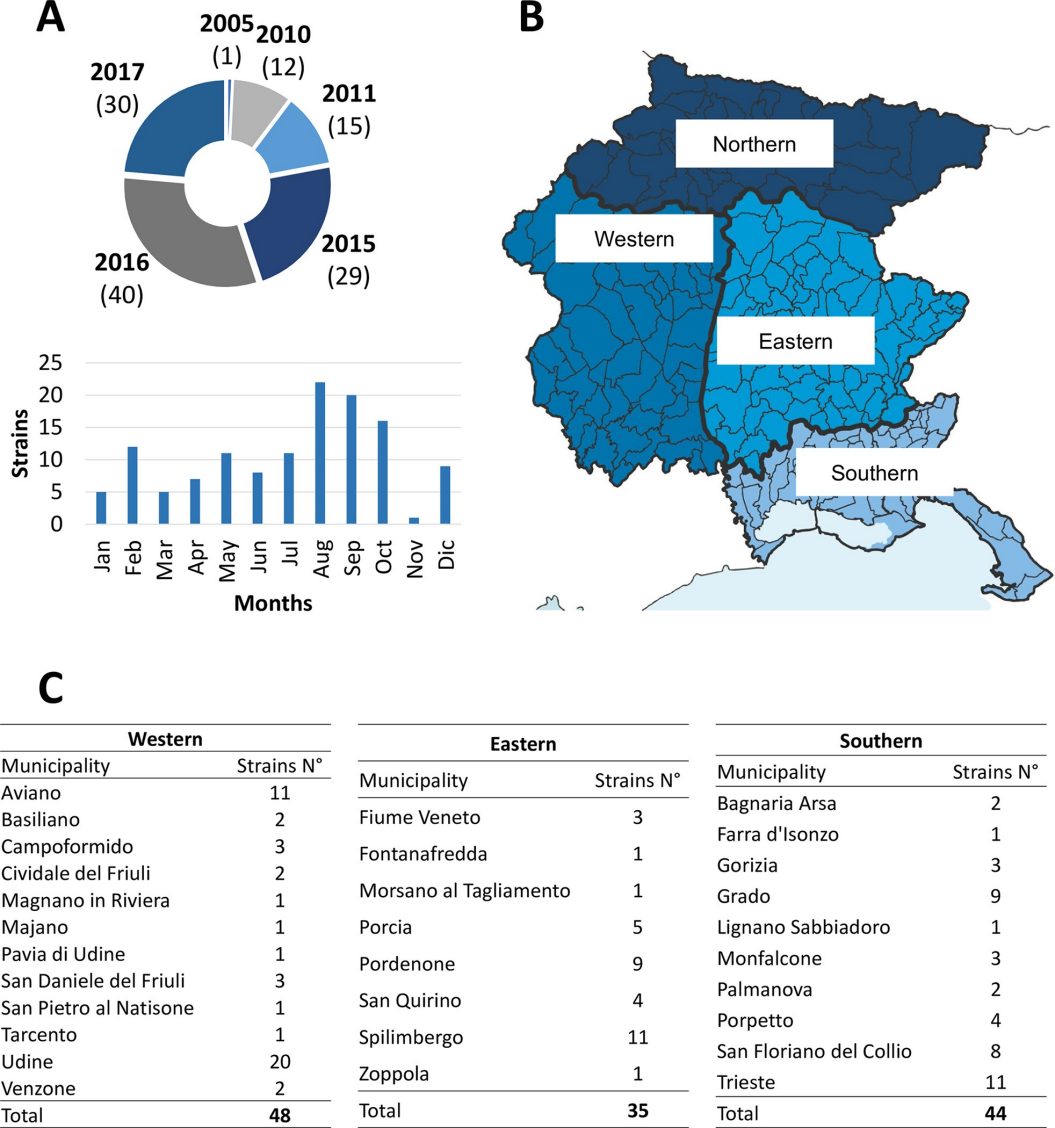
(**A**) Number of strains analysed for each study year and month; (**B**) Climatic subdivision of the Friuli Venezia Giulia region; (**C**) Municipalities included in each subzone, and the number of *L*. *pneumophila* strains isolated in them.

The dataset comprised a total of 127 strains whose isolation time, location, and serogroup were detailed in **S1 Table**. As shown in **Fig 1A**, these strains were isolated across different years, spanning from 2005 to 2017, distributed throughout all the months of the year, with maximum values (22 strains) in August, and minimum values (1 strain) in the month of November. Concerning geographical distribution, as depicted in **Fig 1B**, the strains were classified into four macro zones within the Friuli Venezia Giulia region based on climatic conditions. The northern area, sparsely populated, is characterized by mountainous terrain and a cooler climate. The plain area, where the majority of the population resides, can be further divided into western and eastern areas, separated by the Tagliamento river, the primary watercourse of the region. Lastly, a southern zone, distinguished by a milder climate and lower precipitation near the coast. **Fig 1C** illustrates the various municipalities where the different strains were isolated; notably, out of the 127 total strains, 48 strains were isolated in the western zone, 35 in the eastern zone, and 44 in the southern zone.

### 3.2 Rep-PCR, RAPD, and Sau-PCR profiles analysis

From the genotypic characterization, only Sau-PCR and RAPD techniques resulted effective in providing genetic fingerprints suitable for strain differentiation. Conversely, the primer used for the rep-PCR technique, in many instances, failed to yield genetic characterization profiles deemed adequate.

**Fig 2:**
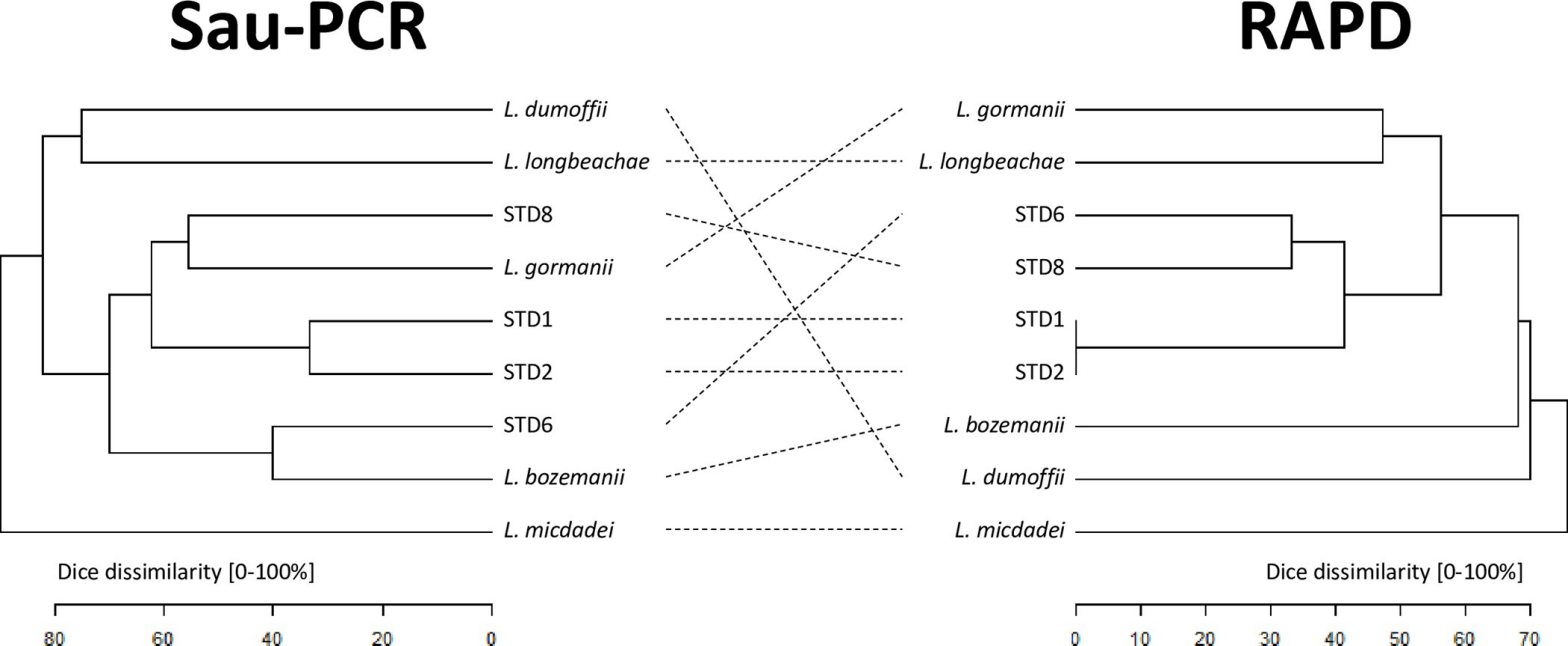
Sau-PCR (left) and RAPD (right) dendrograms, represented as Dice’s dissimilarity matrix, of *Legionella* spp. reference strains.

**Fig 3:**
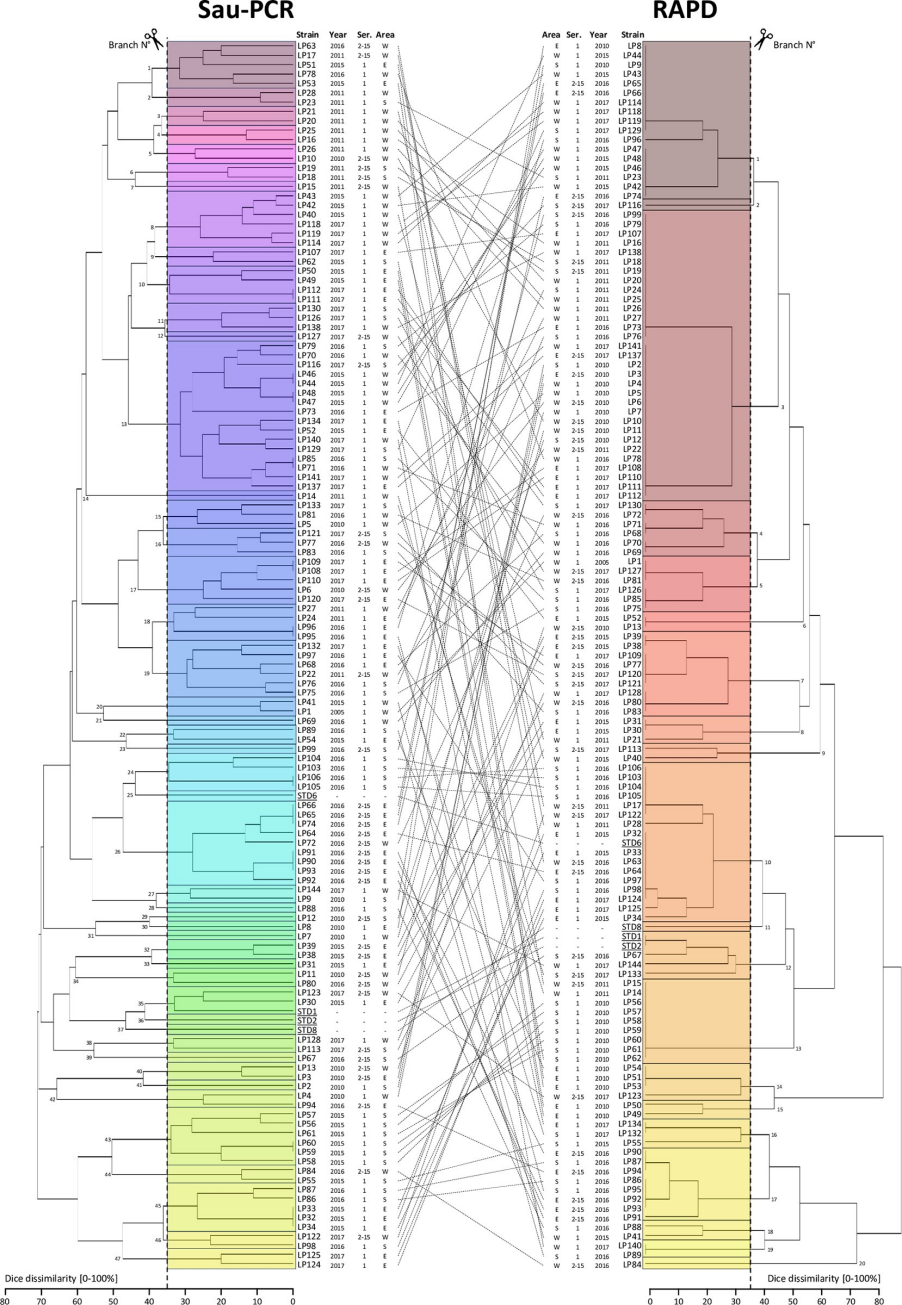
Dice’s dissimilarity matrix calculated for Sau-PCR (left) and RAPD (right) profiles.

Therefore, considering the Sau-PCR and RAPD profiles of the standard strains, significant differences in similarities among the various species analysed emerged in relation to the two different techniques (**Fig 2**). It was possible to observe that, using both methodologies, *L*. *micdadei* resulted the most dissimilar strain compared to others, followed by *L*. *dumoffii* and *L*. *longbeachae*. Conversely, considering the results obtained from Sau-PCR, it was observed that the serotype reference strains of *Lp* did not strictly cluster together. In fact, strains STD1 and STD2 were separated from strain STD8, which showed a higher similarity (44.45%) with *L*. *gormanii*. In the case of strain STD6, a similarity of 60.01% was observed with the *L*. *bozemanii* reference strain. In contrast, from the results obtained from RAPD technique, it was noticeable that all reference strains from different serogroups of *Lp* separated from other *Legionella* spp. with a similarity threshold above 43.83%. Within this cluster, strains STD1 and STD2 showed an identical profile, while differentiating from strains STD8 and STD6 by a dissimilarity percentage above 58.58%. In turn, these latter two strains differentiated at a similarity percentage of 66.67%. Analysing the results of regional query strains in comparison with the reference strains of the four different serogroups of *L*. *pneumophila*, the Sau-PCR demonstrated a higher discriminatory capacity than the RAPD technique. In the obtained dendrograms (**Fig 3**), by applying a dissimilarity cutoff level of 35%, chosen to be more restrictive than the discrimination threshold observed previously among different species, and capable of segregating the various standard strains of different serotypes of *L*. *pneumophila*, Sau-PCR technique distinguished strains into 47 branches with 32 clusters and 15 single strains, whereas the RAPD technique yielded only 20 branches with 17 clusters and 3 single strains. Notably, the Sau-PCR technique identified several strains as clones, forming clusters with higher numerosity. Given these differences in clusters composition, and the fact that in few cases the same strains were clustered together by both techniques, it can be noted how the two techniques assessed the presence of distinct genetic traits and clustered the different strains differently.

### 3.3 Fatty acids composition

**Fig 4:**
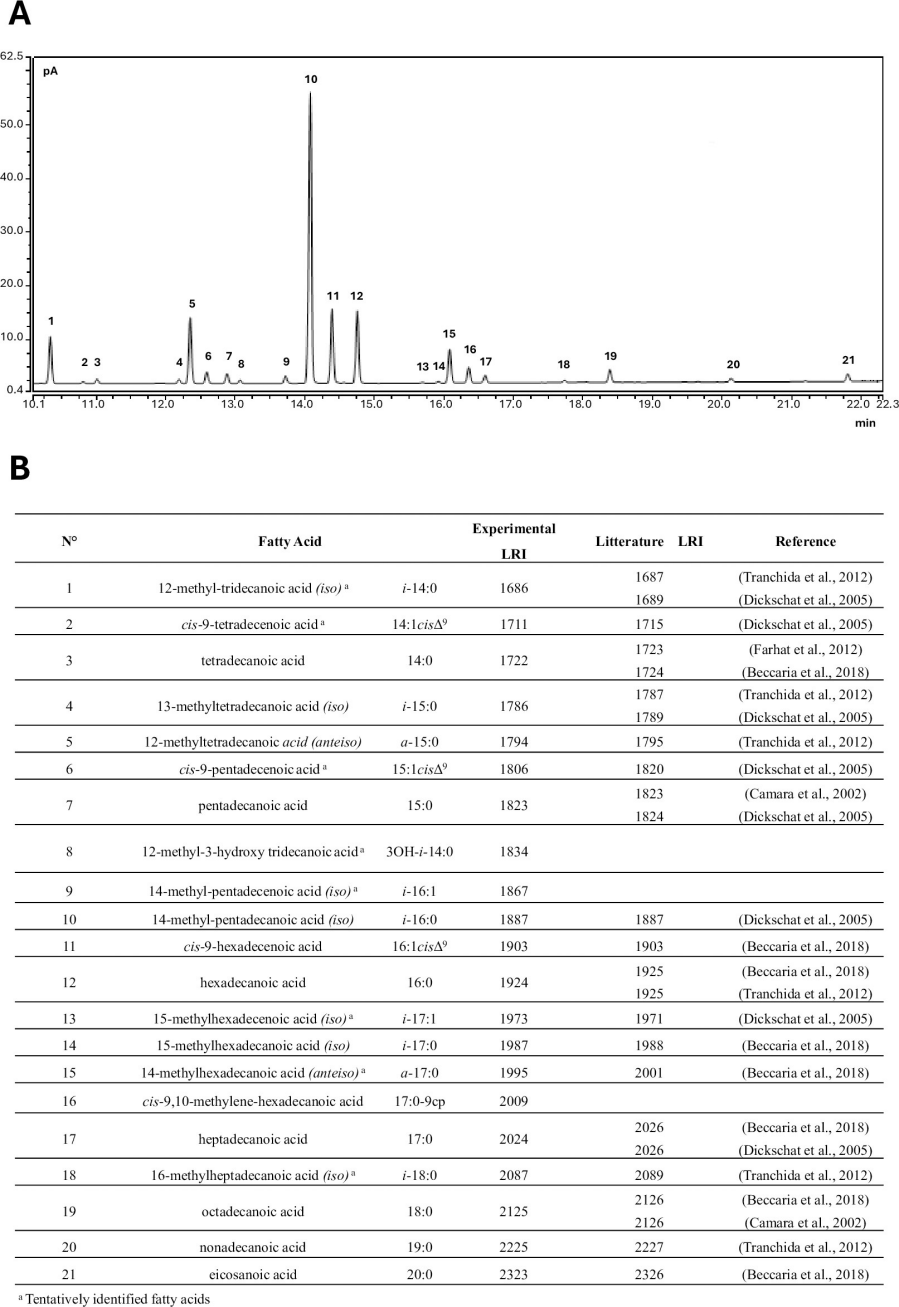
(**A**) Example of GC-FID chromatogram of fatty acid methyl esters from *L*. *pneumophila* strain SG1; (**B**) FAMEs identification from GC-FID chromatograms achieved by comparing retention times and mass spectra with those of BAME Mix, and tentative identification obtained by comparing the linear retention index calculated by injecting a mixture of n-alkanes C7-C30, with literature data and the mass spectra with the mass spectral library NIST 14.

FAMEs identification from GC-FID chromatograms (**Fig 4A**) achieved by comparing retention times and mass spectra with those of BAME standards Mix or tentative identified (**Fig 4B**), identified 21 profiles.

From the subsequent analyses, comparing the fatty acid compositions of reference strains, it was possible to observe the differences between the four *L*. *pneumophila* reference strains and those of others *Legionella* spp. strains (**Table 1**).

**Table 1:** Percentage composition (values > 0.1%) of cellular fatty acids of reference strains of *Legionella* spp. The mean and standard deviation values are reported, followed by letters indicating significantly different groups, evaluated using the Tukey test (p < 0.01).

Fatty Acid	STD 1		STD2		STD 6		STD 8		*L*. *longbeachae*		*L*. *bozemanae*		*L*. *pittsburghensis*		*L*. *dumoffii*		*L*. *gormanii*	
***i*-14:0**	2.89 ± 0.18	bcd	5.48 ± 0.45	a	2.81 ± 0.35	cd	3.46 ± 0.51	bc	3.12 ± 0.11	bc	2.09 ± 0.16	de	0.67 ± 0.04	f	1.49 ± 0.19	ef	3.85 ± 0.04	b
**14:1*cis*Δ** ^**9**^	0.18 ± 0.04	ab	0.29 ± 0.03	a	0.20 ± 0.00	ab	0.20 ± 0.03	ab	0.26 ± 0.00	ab	< 0.1	b	0.14 ± 0.03	b	0.19 ± 0.02	ab	0.10 ± 0.07	b
**14:00**	1.32 ± 0.19	a	1.10 ± 0.01	ab	1.19 ± 0.04	a	1.31 ± 0.01	a	0.84 ± 0.02	bc	0.53 ± 0.03	d	0.47 ± 0.04	d	0.60 ± 0.10	cd	0.53 ± 0.05	d
***i*-15:0**	0.54 ± 0.07	b	0.48 ± 0.02	bc	0.39 ± 0.02	c	0.82 ± 0.01	a	0.22 ± 0.00	d	0.16 ± 0.01	d	0.50 ± 0.01	b	0.21 ± 0.01	d	0.18 ± 0.05	d
***a*-15:0**	11.50 ± 0.89	d	9.74 ± 0.22	d	9.22 ± 0.79	d	10.97 ± 0.63	d	9.98 ± 0.10	d	27.4 ± 1.6	b	36.8 ± 0.44	a	30.61 ± 2.26	b	20.36 ± 0.38	c
**15:1*cis*Δ** ^**9**^	0.98 ± 0.11	e	2.19 ± 0.18	a	0.97 ± 0.17	e	1.19 ± 0.16	de	1.73 ± 0.03	bc	1.47 ± 0.11	cd	1.56 ± 0.10	cd	0.13 ± 0.09	f	1.99 ± 0.04	ab
**15:00**	2.12 ± 0.07	de	2.48 ± 0.05	c	1.89 ± 0.04	e	2.50 ± 0.20	c	2.20 ± 0.02	cde	3.45 ± 0.14	a	2.34 ± 0.06	cd	0.29 ± 0.05	f	3.06 ± 0.03	b
**3OH-*i-*14:0**	0.66 ± 0.12	a	0.67 ± 0.09	a	0.52 ± 0.05	a	0.63 ± 0.06	a	< 0.1	c	< 0.1	c	0.22 ± 0.00	b	< 0.1	c	< 0.1	c
***i*-16:1**	0.68 ± 0.09	c	1.02 ± 0.06	ab	0.75 ± 0.04	c	0.82 ± 0.10	bc	0.29 ± 0.03	de	0.18 ± 0.03	de	1.11 ± 0.01	a	0.36 ± 0.07	d	< 0.1	e
***i*-16:0**	25.65 ± 1.53	b	34.21 ± 0.36	a	26.45 ± 1.65	b	26.85 ± 2.08	b	15.07 ± 0.58	cde	9.90 ± 0.41	e	13.69 ± 0.28	de	11.78 ± 0.73	de	18.26 ± 0.29	c
**16:1*cis*Δ** ^**9**^	15.74 ± 1.26	b	13.37 ± 0.23	bc	15.12 ± 0.58	b	13.46 ± 0.36	bc	26.62 ± 0.96	a	12.11 ± 0.31	c	5.89 ± 0.23	d	11.00 ± 1.47	c	11.38 ± 0.28	c
**16:00**	22.87 ± 0.68	a	14.73 ± 1.15	c	22.84 ± 1.82	a	22.73 ± 2.25	a	23.57 ± 0.74	a	19.82 ± 1.86	ab	5.94 ± 0.16	d	16.28 ± 1.32	bc	13.34 ± 0.43	c
***i*-17:1**	0.11 ± 0.08	b	0.11 ± 0.01	b	0.18 ± 0.01	b	0.17 ± 0.01	b	< 0.1	b	0.16 ± 0.01	b	3.25 ± 0.02	a	< 0.1	b	0.14 ± 0.13	b
***i*-17:0**	0.36 ± 0.01	bc	0.30 ± 0.00	c	0.33 ± 0.01	bc	0.60 ± 0.07	a	0.37 ± 0.01	bc	0.63 ± 0.03	a	0.31 ± 0.01	c	0.47 ± 0.10	ab	0.54 ± 0.05	a
***a*-17:0**	7.23 ± 0.41	de	4.83 ± 0.09	e	6.88 ± 0.34	de	6.69 ± 0.54	de	5.74 ± 0.37	e	9.53 ± 0.52	cd	22.63 ± 0.68	a	17.44 ± 2.43	b	10.54 ± 0.17	c
**17:0-9cp**	0.45 ± 0.12	e	2.08 ± 0.15	cd	1.15 ± 0.44	de	0.88 ± 0.09	e	2.83 ± 0.11	bc	3.33 ± 0.59	b	2.15 ± 0.11	cd	5.11 ± 0.51	a	5.39 ± 0.31	a
**17:00**	1.34 ± 0.16	c	1.72 ± 0.04	bc	1.61 ± 0.06	c	1.51 ± 0.13	c	2.23 ± 0.22	b	5.01 ± 0.15	a	0.76 ± 0.03	d	0.76 ± 0.09	d	5.29 ± 0.32	a
***i*-18:0**	0.29 ± 0.01		0.33 ± 0.01		0.32 ± 0.04		0.30 ± 0.03		0.30 ± 0.04		0.19 ± 0.02		0.16 ± 0.02		0.33 ± 0.08		0.30 ± 0.21	
**18:00**	3.2 ± 0.38	ab	2.37 ± 0.10	abc	3.78 ± 0.82	a	3.12 ± 0.72	ab	2.47 ± 0.25	abc	1.91 ± 0.26	bc	0.20 ± 0.01	d	2.05 ± 0.59	bc	1.45 ± 0.15	cd
**19:00**	0.62 ± 0.15	bc	0.87 ± 0.06	ab	0.84 ± 0.18	b	0.65 ± 0.12	bc	0.43 ± 0.07	cd	0.97 ± 0.06	ab	0.14 ± 0.04	de	< 0.1	e	1.24 ± 0.15	a
**20:00**	1.35 ± 0.40	ab	1.51 ± 0.12	ab	2.40 ± 0.66	a	1.31 ± 0.32	b	0.55 ± 0.11	b	0.52 ± 0.08	b	0.83 ± 0.22	b	0.56 ± 0.22	b	0.54 ± 0.06	b

The prevalence of α-15:0 fatty acid was notable in *L*. *bozemanii* (27.4 ± 1.6%), *L*. *micdadei* (36.8 ± 0.44%), *L*. *gormanii* (20.36 ± 0.38%), and *L*. *dumoffii* (30.61 ± 2.26%). Conversely, in accordance with prior observations, *L*. *longbeachae* exhibited a higher percentage of the unsaturated fatty acid 16:1cisΔ9 (26.62 ± 0.96%) [40, 41].

*L*. *micdadei* was characterized by a higher percentage of branched α-17:0 fatty acid (22.63 ± 0.68%) and iso-17:1 fatty acid (3.25 ± 0.02%), while displaying a lower percentage of 16:0 fatty acid (5.94 ± 0.16%).

**Fig 5:**
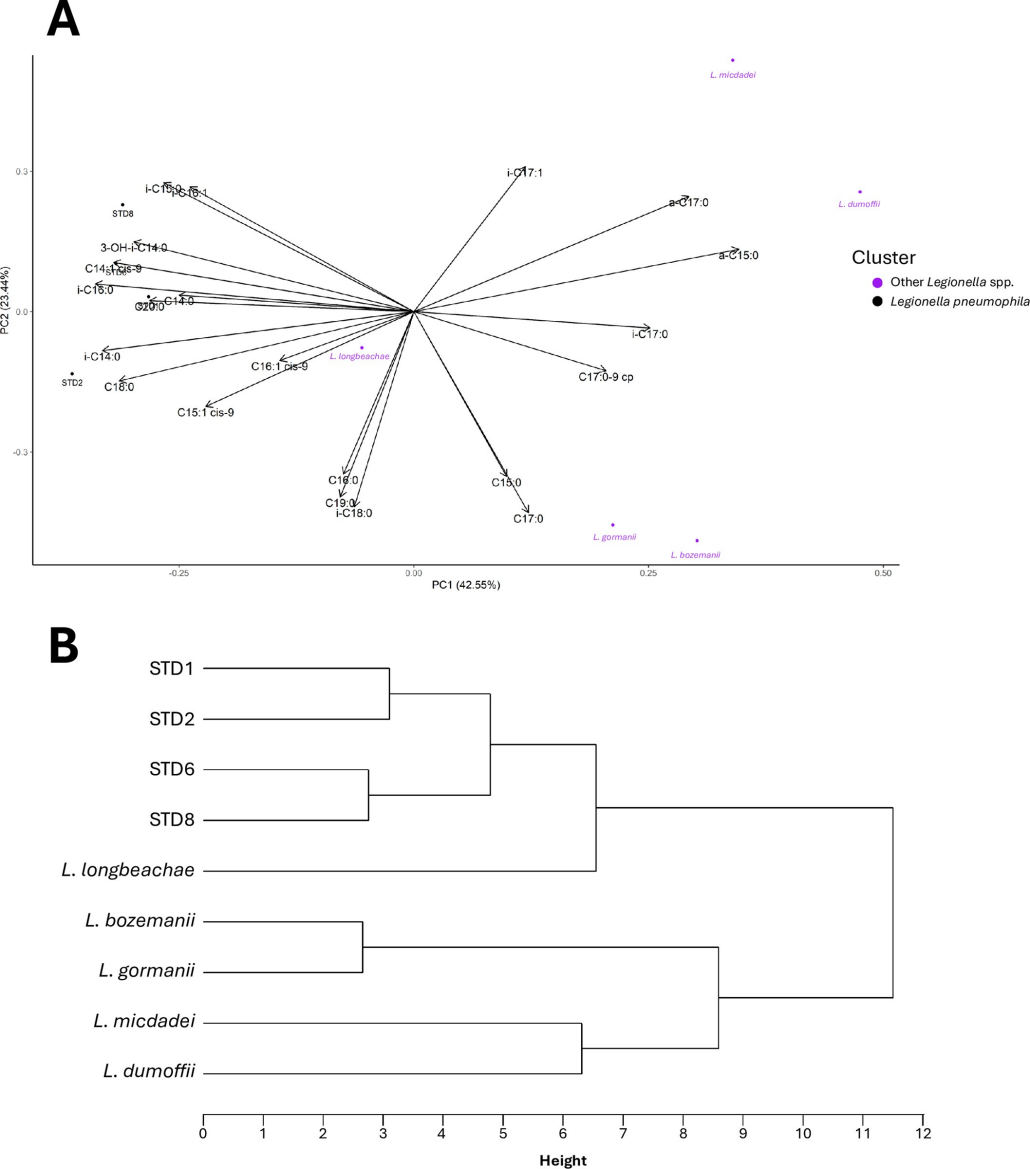
(**A**) PCA analysis of the strains based on their fatty acid composition; (**B**) Hierarchical Cluster Analysis of the reference strains measured using Ward’s minimum variance method. The height of the fusion provided on the horizontal axis indicates the dissimilarity between two strains.

Notably, *L*. *pneumophila* strains were characterized by significantly higher presence of iso-16:0 fatty acid in comparison to the others considered *Legionella* spp. strains. A better visualization of the correlation between the strains and the fatty acids percentages could be observed in the PCA analysis reported in **Fig 5A**. Furthermore, the hierarchical cluster analysis reported in **Fig 5B** provided a detailed view, revealing that the four different serotypes of *L*. *pneumophila* exhibited a similarity height >95% among themselves, differing by more than 6.5% from *L*. *longbeachae* that resulted the most similar strain. The other species showed greater disparities, with *L*. *bozemanii* and *L*. *gormanii* sharing a similarity height of approximately 97% but differing by just under 9% from *L*. *micdadei* and *L*. *dumoffii*, which, in turn, shared a similarity height of about 6.5%.

**Fig 6:**
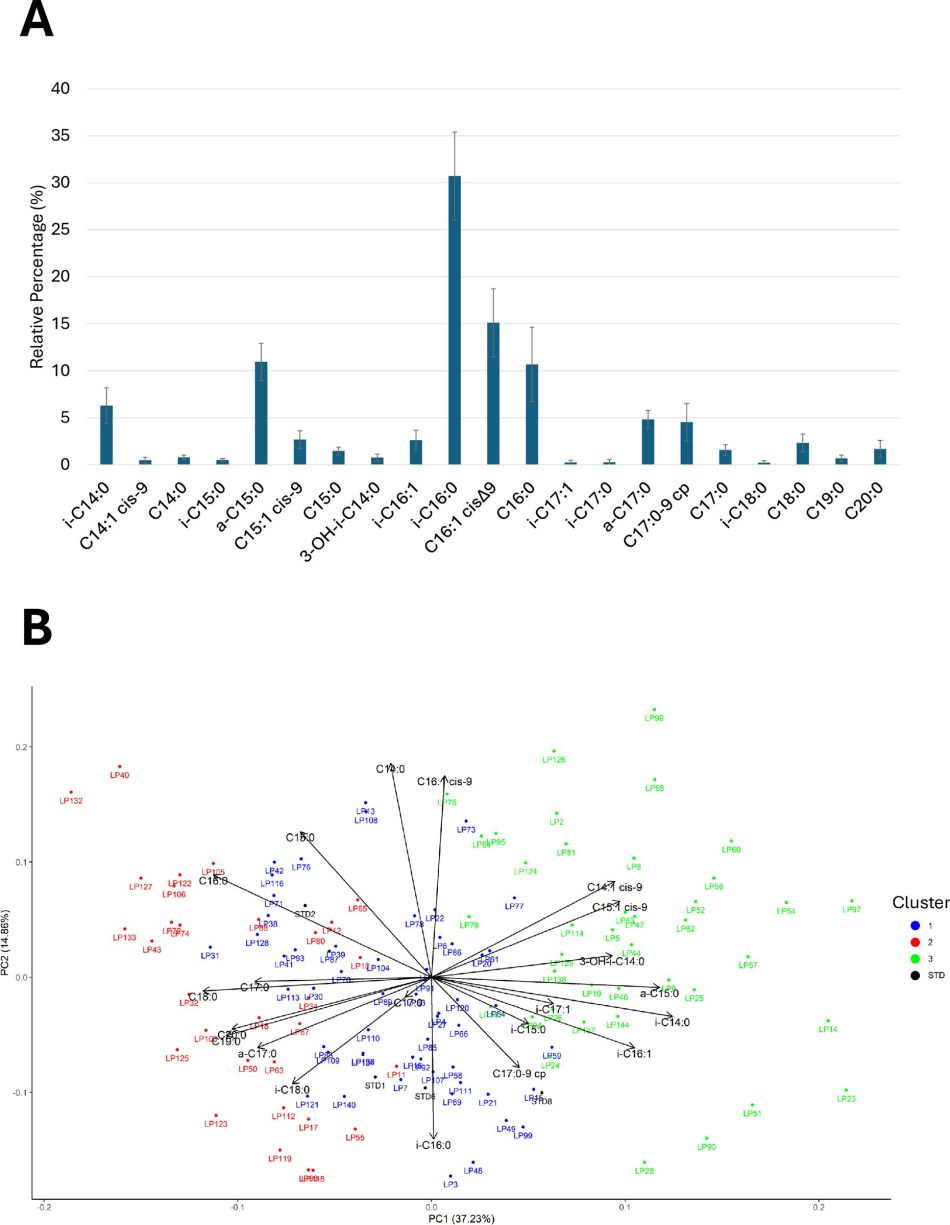
(**A**) Mean relative percentage of fatty acids composition the query strains; (**B**) PCA analysis of the strains based on their fatty acid composition.

Examining in detail the regional *L*. *pneumophila* strains, a fatty acid profile comparable to that of the previously described *L*. *pneumophila* reference strains was observed (**S2 Table**). Indeed, a composition characterized by fatty acids with both even and odd carbon chain lengths ranging from 14 to 21 carbon atoms was detected (**Fig 6A**). The quantitatively most abundant fatty acids were branched, with i-16:0 alone constituting 30.70 ± 4.68%, followed by C16:1 cisΔ9 (15.11 ± 3.61%), *a*-C15:0 (10.94 ± 1.98%), a-17:0 (4.83 ± 0.95%), together comprising over 60% of the identified fatty acids for each strain. These fatty acids are in fact considered the primary markers for a preliminary differentiation of different *Legionella* species. In addition to 16:1 cisΔ9 acid, considering the 16-carbon chain fatty acids, 16:0 acid was present at a significant percentage (10.67 ± 3.96%). Also, the presence of 17:0-9cp acid (4.52 ± 2.00%), and 3-OH-*i*-14:0 hydroxy acid (0.77 ± 0.38%) were identified. Analysing the differences in fatty acid composition in relation to the serotype, no clear differences were observed among standard strains of different serogroups, both among themselves and in relation to the average values of the 127 analysed *Lp* strains. In particular, observing the PCA plot (**Fig 6B**), a high similarity was evident among STD1, STD6, and STD8 strains, while the strain STD2 resulted the only strain that clearly differentiated from the others, mainly due to a higher percentage of the fatty acid C16:0.

**Fig 7:**
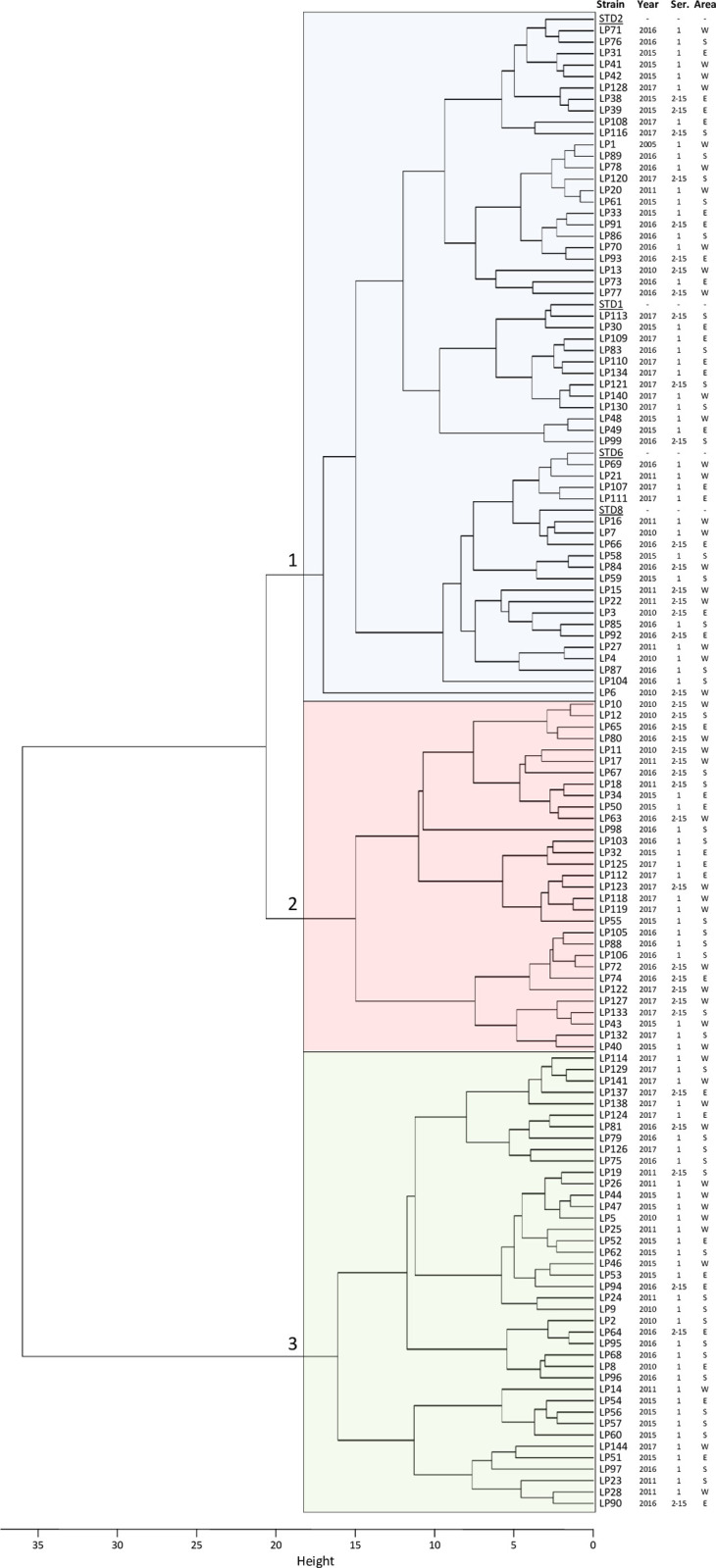
Hierarchical cluster analysis of the analysed strains measured using Ward’s minimum variance method. The height of the fusion provided on the horizontal axis indicates the dissimilarity between two strains.

From the hierarchical cluster analysis, it was possible to observe the relationships between different strains based on fatty acids (**Fig 7**). As also detected through the Gap Statistic Method [42], it was possible to identify three main clusters (distinguished with the same colours also in the PCA plot of **Fig 6B**). At a dissimilarity height exceeding 35%, 40 strains separated (cluster 3, represented in green), which were the most dissimilar from the others, characterized by higher percentages of i-C14:0, C14:1 cisΔ9, *a*-C15:0, C15:1 cisΔ9, 3-OH-*i*-C14:0, and i-C16:1 fatty acids. More similar were the other two clusters (cluster 1, highlighted by blue colour, grouping 56 strains, and cluster 2, highlighted by red colour, collecting 31 strains), which separate from each other at a dissimilarity height of about 20%. Among these, all standard strains of different serogroups grouped in cluster 1, while cluster 2 was found to be the most dissimilar from cluster 3 and composed of strains characterized by a higher presence of C16:0, a-C17:0, C17:0, i-C18:0, C18:0, C19:0, and C20:0.

### 3.4 Obtained cluster analysis

**Fig 8:**
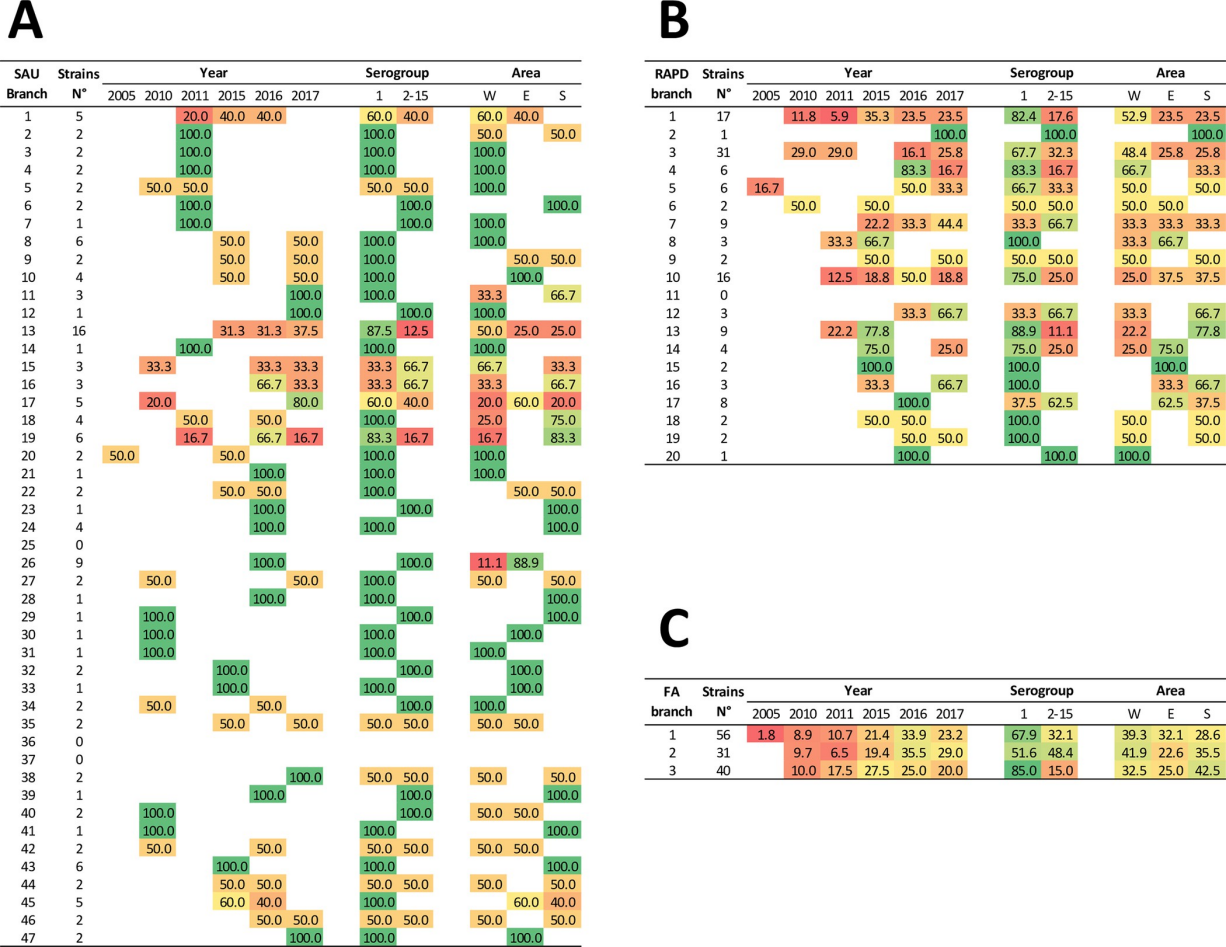
Composition of the clusters obtained from Sau-PCR (**A**), RAPD (**B**) and Fatty Acids (**C**) analyses in relation to the year, serogroup, and geographical area of isolation. In each cell the number of strains (Strains N°) of each cluster is reported as a percentage value. DSMZ reference strains are not considered.

Analysing the different clusters obtained from various techniques (whose branch code number for each of the techniques applied is reported in **S1 Table**) it was possible to observe how they were subdivided based on year, geographic area, and serogroup. Beginning with the results obtained from the Sau-PCR technique (**Fig 8A**), it demonstrated to be the technique with the highest discriminatory power, resulting in the highest number of branches obtained. Excluding reference strains, considering only the regional strains were divided into 44 branches, of which 27.3% consisted of single strains, 40.9% of two strains, and only the remaining 31.8% of more than three. Due to the high level of discrimination, it was therefore possible to observe a high correspondence of strains within each branch regarding the serogroup and a good correspondence regarding the year of isolation, while the correlation to the isolation area resulted weaker. Of particular interest in this case were the larger clusters. Particularly, it was observed that in branches No. 1, No. 13, No. 17, No. 18, No. 19, and No. 45, strains from different years, different areas, and even discordant serogroups grouped together. Conversely, branch No. 8 presented strains from the same area and serogroup but from two distinct years, suggesting persistence of this strain over time. Similarly, branch No. 26 was clustered strains of the same serogroup isolated in the same year but from different areas. Lastly, branch No. 45 presented strains isolated in different years, from different areas, but all belonging to serogroup I.

Turning to the results obtained from RAPD analysis, the regional strains were divided into 19 branches. In this case, no clear correlations were found between the branches and the investigated variables. Similar considerations can be made for the clusters obtained from fatty acids analysis. Indeed, it was not possible to observe a clear correlation between the strains and year and place of isolation, while concerning the serogroup, only cluster No. 3 showed a predominance of strains belonging to serogroup 1. It should also be noted that the absence of correlation cannot be attributed to the cluster selection threshold, as observable in **Fig 7**, this lack of relationships is maintained even at much higher cutoff levels.

## 4 Discussion

### 4.1 Characteristics of the strain collection

The strain collection preserved by ARPA FVG considered in this work consisted of 127 strains isolated over a period of 12 years throughout the regional territory of Friuli Venezia Giulia in northeastern Italy. The strains were evenly distributed among the different zones into which the region could be divided, except for the northern zone, where both the colder climate and lower population density contribute to reducing the incidence of *Lp* presence in water systems [43]. Furthermore, the number of strains in the dataset followed a temporal distribution observed in larger datasets from this region, with periods of maximum risk of *Lp* presence at the end of summer (August–November) and a decrease in winter and spring months [44]. Therefore, given its size and uniform spatio-temporal distribution, the dataset can be considered adequate for representing the spread of this pathogen in the region.

### 4.2 Genetic insights

The initial analyses conducted focused on verifying genetic patterns. For this purpose, rep-PCR, RAPD, and Sau-PCR techniques were used, but only the latter two provided useful profiles for strain comparison. Despite rep-PCR being reported in the literature as effective in discriminating *Legionella* spp. [45], the primers and conditions used in this study did not yield comparable profiles. On the contrary, RAPD and Sau-PCR proved effective in providing discriminative profiles of the different strains. These techniques are reported to be more suitable for differentiating *Lp* strains compared to other techniques such as pulsed field gel electrophoresis [16]. By applying a cutoff level of 35% on the dendrograms obtained from both techniques, a much higher discrimination was observed with Sau-PCR compared to the RAPD technique. Despite the high discriminatory capacity observed, very few studies have employed this technique to analyse this pathogen [16]. When comparing how the strains were divided and clustered from these two analytical techniques, there was poor congruence, and in most cases, the strains showed different relationships with each other. However, these techniques target different genetic characteristics and have different levels of stringency. Such discrepancies in clustering have also been observed in other similar studies [46], demonstrating the relevance of comparing different techniques for this type of study. Additionally, no clear clustering pattern was observed regarding serogroup, which could have been influenced by the primers used [47]. However, the primary purpose of this work was not to identify primers suitable for species identification or serogroup division but to identify the presence of potential clones in different locations and periods and to verify the presence of correlation with origins. In this regard, it was possible to observe a ubiquitous geographical distribution of the different clusters, suggesting spread throughout the region rather than specific locations of certain clusters. These observations align with several studies, where a very high genetic variability of *Lp* strains has been observed [48, 49], with genetically different strains found in the same area [50]. Moreover, as observed in other studies, no correlation was found between geographical origin and genetic characteristics [51].

### 4.3 Phenotypic insights

These genotypic observations were also supported by phenotypic analysis of fatty acids, where no clear correlations were identified based on geographic area and year of isolation. In this case, as observable in the PCA, regional *Lp* strains distinguished themselves from other analysed species by a higher association with fatty acids i-C15:0, i-C16:1, 3-OH-i-C14:0, C14:1 cis-9, iC16:0, C14:0, C20:0, i-C14:0, C18:0, C16:1 cis-9, C15:1 cis9. Moreover, these strains showed a fatty acid profile comparable to that previously described for *L*. *pneumophila* by earlier authors [31, 32, 52], with a high presence of i-C16:0 [41, 52, 53]. As reported in the literature [52], 17:0-9cp acid, containing a cyclopropane ring in the carbon chain, and 3-OH-i-14:0 hydroxy acid were also identified. This fatty acid, also highlighted by other studies on *Lp* strains analysed through basic hydrolysis [41], is the only hydroxylated fatty acid identified with the sample preparation method used in this study. Despite its low presence, it proves important in the study of *Legionella* spp. Hydroxylated fatty acids at positions 2 and 3, both linear and branched, have been primarily detected in studies on the lipopolysaccharides of the cell membrane of this bacterial species. These fatty acids are specifically present in the hydrophobic portion of the lipopolysaccharide, known as Lipid A, to which they are linked by an amide bond and have often been found as distinctive features unique to *Lp* strains [53–55]. However, it should be noted that different sample preparation methods using basic hydrolysis (as in this study) or acidic hydrolysis release different types of fatty acids. In a study conducted on *Legionella lytica*, hydroxylated fatty acids of lipopolysaccharides, characterized by a strong amide bond, were determined only after hydrolysis with 4 M HCl at 100°C for six hours, while they were generally not detected by basic hydrolysis [56]. Additionally, other studies report that acidic hydrolysis degrades cyclopropane acids, forming artifacts that may interfere with gas chromatographic analysis [57]. Instead, the regional *Lp* strains considered in this work could be divided into three main clusters according to their fatty acid composition. The first cluster (cluster 1), which included reference strains of different serogroups, was characterized by strains with intermediate relative percentages of all FAs. Conversely, a second cluster (cluster 2) was characterized by a higher presence of C16:0, C17:0, C18:0, C20:0, C19:0, a-C17:0, i-C18:0, and a third cluster (cluster 3) was characterized by a higher content of C14:1 cis-9, C15:1 cis-9, 3-OH-i-C14:0, a-C15:0, i-C14:0, and i-C16:1.

## 5 Conclusions

This study has revealed a high genetic differentiation of *Lp* strains isolated in Friuli Venezia Giulia, showing that the various genotypic and phenotypic traits are not associated with specific locations and times of isolation, but rather there is a widespread distribution across the entire territory and over time. Indeed, in addition to not observing a clear correlation of the strains with the macro areas identified, it was possible to observe a high differentiation within the same municipality, with strains with the same genotypic/phenotypic characteristics present in municipalities very distant from each other. Furthermore, the conducted analysis highlighted the effectiveness of the Sau-PCR technique in characterizing *Legionella* spp., a technique still scarcely employed for analysing this important pathogen.

## Supporting information

S1 Table Detailed description of the dataset metadata. (DOCX)

S2 Table Fatty acids composition of the different isolated *Lp* strains. (DOCX)
