# Supplementary material for: Genotypic and phenotypic profiling of 127 Legionella pneumophila strains: Insights into regional spread
Source: PLoS One. 2024 Jul 19;19(7):e0307646. doi: 10.1371/journal.pone.0307646 (PMC11259292; doi:10.1371/journal.pone.0307646)
Supplement: S1 Table — (DOCX) [file pone.0307646.s001.docx]

**Table S1.** Detailed description of the Dataset metadata

| **Complete strain name** | | **A short name** | | **Isolation Municipality** | | **Zone** | | **Collection Date** | | | | **Predicted**  **Serotype** | | **Branch** | | | | | |
| --- | --- | --- | --- | --- | --- | --- | --- | --- | --- | --- | --- | --- | --- | --- | --- | --- | --- | --- | --- |
|  |  |  |  |  |  |  |  | **Month** | | **Year** | |  |  | **Sau-PCR** | | **RAPD** | | **FA** | |
| LP1_2005_3688 | | LP1 | | San Daniele del Friuli | | W | | May | | 2005 | | 1 | | 20 | | 5 | | 1 | |
| LP2_2010_7035 | | LP2 | | Gorizia | | S | | Jun | | 2010 | | 1 | | 41 | | 3 | | 3 | |
| LP3_2010_7343 | | LP3 | | Morsano al Tagliamento | | E | | Jul | | 2010 | | 8 | | 40 | | 3 | | 1 | |
| LP4_2010_12257 | | LP4 | | Udine | | W | | Oct | | 2010 | | 1 | | 42 | | 3 | | 1 | |
| LP5_2010_12508 | | LP5 | | Udine | | W | | Oct | | 2010 | | 1 | | 15 | | 3 | | 3 | |
| LP6_2010_13100 | | LP6 | | Udine | | W | | Nov | | 2010 | | 11 | | 17 | | 3 | | 1 | |
| LP7_2010_13335 | | LP7 | | Majano | | W | | Dic | | 2010 | | 1 | | 31 | | 3 | | 1 | |
| LP8_2010_13575 | | LP8 | | Pordenone | | E | | Jan | | 2010 | | 1 | | 30 | | 1 | | 3 | |
| LP9_2010_13639 | | LP9 | | Monfalcone | | S | | Feb | | 2010 | | 1 | | 27 | | 1 | | 3 | |
| LP10_2010_14262 | | LP10 | | Udine | | W | | Dic | | 2010 | | 6 | | 5 | | 3 | | 2 | |
| LP11_2010_14388 | | LP11 | | Cividale del Friuli | | W | | Dic | | 2010 | | 8 | | 34 | | 3 | | 2 | |
| LP12_2010_14946 | | LP12 | | Trieste | | S | | Dic | | 2010 | | 6 | | 29 | | 3 | | 2 | |
| LP13_2010_15075 | | LP13 | | Udine | | W | | Dic | | 2010 | | 8 | | 40 | | 6 | | 1 | |
| LP14_2011_1360 | | LP14 | | Udine | | W | | Feb | | 2011 | | 1 | | 14 | | 13 | | 3 | |
| LP15_2011_2680 | | LP15 | | Udine | | W | | Mar | | 2011 | | 7 | | 7 | | 13 | | 1 | |
| LP16_2011_2730 | | LP16 | | San Daniele del Friuli | | W | | Mar | | 2011 | | 1 | | 4 | | 3 | | 1 | |
| LP17_2011_4965 | | LP17 | | Udine | | W | | May | | 2011 | | 8 | | 1 | | 10 | | 2 | |
| LP18_2011_5016 | | LP18 | | Trieste | | S | | May | | 2011 | | 6 | | 6 | | 3 | | 2 | |
| LP19_2011_5017 | | LP19 | | Trieste | | S | | May | | 2011 | | 8 | | 6 | | 3 | | 3 | |
| LP20_2011_5054 | | LP20 | | Udine | | W | | May | | 2011 | | 1 | | 3 | | 3 | | 1 | |
| LP21_2011_6872 | | LP21 | | San Daniele del Friuli | | W | | Jun | | 2011 | | 1 | | 3 | | 8 | | 1 | |
| LP22_2011_7143 | | LP22 | | Udine | | W | | Jul | | 2011 | | 8 | | 19 | | 3 | | 1 | |
| LP23_2011_7324 | | LP23 | | Monfalcone | | S | | Jul | | 2011 | | 1 | | 2 | | 1 | | 3 | |
| LP24_2011_7512 | | LP24 | | Trieste | | S | | Jul | | 2011 | | 1 | | 18 | | 3 | | 3 | |
| LP25_2011_7660 | | LP25 | | Udine | | W | | Jul | | 2011 | | 1 | | 4 | | 3 | | 3 | |
| LP26_2011_7708 | | LP26 | | Cividale del Friuli | | W | | Jul | | 2011 | | 1 | | 5 | | 3 | | 3 | |
| LP27_2011_7798 | | LP27 | | Magnano in Riviera | | W | | Jul | | 2011 | | 1 | | 18 | | 3 | | 1 | |
| LP28_2011_9951 | | LP28 | | Tarcento | | W | | Sep | | 2011 | | 1 | | 2 | | 10 | | 3 | |
| LP30_2015_4925 | | LP30 | | Pordenone | | E | | Apr | | 2015 | | 1 | | 35 | | 8 | | 1 | |
| LP31_2015_4961 | | LP31 | | Pordenone | | E | | Apr | | 2015 | | 1 | | 33 | | 8 | | 1 | |
| LP32_2015_6923 | | LP32 | | Fiume Veneto | | E | | May | | 2015 | | 1 | | 45 | | 10 | | 2 | |
| LP33_2015_6924 | | LP33 | | Fiume Veneto | | E | | May | | 2015 | | 1 | | 45 | | 10 | | 1 | |
| LP34_2015_6925 | | LP34 | | Fiume Veneto | | E | | May | | 2015 | | 1 | | 45 | | 10 | | 2 | |
| LP38_2015_9504 | | LP38 | | Spilimbergo | | E | | Jul | | 2015 | | 6 | | 32 | | 7 | | 1 | |
| LP39_2015_9507 | | LP39 | | Spilimbergo | | E | | Jul | | 2015 | | 6 | | 32 | | 7 | | 1 | |
| LP40_2015_11509 | | LP40 | | Aviano | | W | | Sep | | 2015 | | 1 | | 8 | | 9 | | 2 | |
| LP41_2015_11525 | | LP41 | | Aviano | | W | | Sep | | 2015 | | 1 | | 20 | | 18 | | 1 | |
| LP42_2015_11526 | | LP42 | | Aviano | | W | | Sep | | 2015 | | 1 | | 8 | | 1 | | 1 | |
| LP43_2015_11527 | | LP43 | | Aviano | | W | | Sep | | 2015 | | 1 | | 8 | | 1 | | 2 | |
| LP44_2015_11755 | | LP44 | | Aviano | | W | | Sep | | 2015 | | 1 | | 13 | | 1 | | 3 | |
| LP45_2015_11756 | | LP46 | | Aviano | | W | | Sep | | 2015 | | 1 | | 13 | | 1 | | 3 | |
| LP46_2015_11762 | | LP47 | | Aviano | | W | | Sep | | 2015 | | 1 | | 13 | | 1 | | 3 | |
| LP47_2015_11763 | | LP48 | | Aviano | | w | | Sep | | 2015 | | 1 | | 13 | | 1 | | 1 | |
| LP48_2015_11765 | | LP49 | | Spilimbergo | | E | | Sep | | 2015 | | 1 | | 10 | | 15 | | 1 | |
| LP49_2015_12212 | | LP50 | | Spilimbergo | | E | | Sep | | 2015 | | 1 | | 10 | | 15 | | 2 | |
| LP50_2015_12213 | | LP51 | | Pordenone | | E | | Oct | | 2015 | | 1 | | 1 | | 14 | | 3 | |
| LP51_2015_12754 | | LP52 | | Pordenone | | E | | Oct | | 2015 | | 1 | | 13 | | 6 | | 3 | |
| LP52_2015_12755 | | LP53 | | Pordenone | | E | | Oct | | 2015 | | 1 | | 1 | | 14 | | 3 | |
| LP53_2015_12756 | | LP54 | | Pordenone | | E | | Oct | | 2015 | | 1 | | 22 | | 14 | | 3 | |
| LP54_2015_12757 | | LP55 | | San Floriano del Collio | | S | | Oct | | 2015 | | 1 | | 44 | | 16 | | 2 | |
| LP56_2015_13149 | | LP56 | | San Floriano del Collio | | S | | Oct | | 2015 | | 1 | | 43 | | 13 | | 3 | |
| LP57_2015_13150 | | LP57 | | San Floriano del Collio | | S | | Oct | | 2015 | | 1 | | 43 | | 13 | | 3 | |
| LP58_2015_13151 | | LP58 | | San Floriano del Collio | | S | | Oct | | 2015 | | 1 | | 43 | | 13 | | 1 | |
| LP59_2015_13152 | | LP59 | | San Floriano del Collio | | S | | Oct | | 2015 | | 1 | | 43 | | 13 | | 1 | |
| LP60_2015_13153 | | LP60 | | San Floriano del Collio | | S | | Oct | | 2015 | | 1 | | 43 | | 13 | | 3 | |
| LP61_2015_13154 | | LP61 | | San Floriano del Collio | | S | | Oct | | 2015 | | 1 | | 43 | | 13 | | 1 | |
| LP62_2015_13155 | | LP62 | | San Floriano del Collio | | S | | Oct | | 2015 | | 1 | | 9 | | 13 | | 3 | |
| LP63_2016_1544 | | LP63 | | Pavia di Udine | | W | | Feb | | 2016 | | 8 | | 1 | | 10 | | 2 | |
| LP64_2016_1756 | | LP64 | | Spilimbergo | | E | | Feb | | 2016 | | 3 | | 26 | | 10 | | 3 | |
| LP65_2016_1763 | | LP65 | | Spilimbergo | | E | | Feb | | 2016 | | 3 | | 26 | | 1 | | 2 | |
| LP66_2016_1764 | | LP66 | | Spilimbergo | | E | | Feb | | 2016 | | 3 | | 26 | | 1 | | 1 | |
| LP67_2016_1798 | | LP67 | | Trieste | | S | | Feb | | 2016 | | 6 | | 39 | | 12 | | 2 | |
| LP68_2016_1801 | | LP68 | | Trieste | | S | | Feb | | 2016 | | 1 | | 19 | | 4 | | 3 | |
| LP69_2016_2458 | | LP69 | | Udine | | W | | Feb | | 2016 | | 1 | | 21 | | 4 | | 1 | |
| LP70_2016_2460 | | LP70 | | Udine | | W | | Feb | | 2016 | | 1 | | 13 | | 4 | | 1 | |
| LP71_2016_4320 | | LP71 | | Udine | | W | | Apr | | 2016 | | 1 | | 13 | | 4 | | 1 | |
| LP72_2016_4381 | | LP72 | | Udine | | W | | Apr | | 2016 | | 6 | | 26 | | 4 | | 2 | |
| LP73_2016_4407 | | LP73 | | Spilimbergo | | E | | Apr | | 2016 | | 1 | | 13 | | 3 | | 1 | |
| LP74_2016_4429 | | LP74 | | Spilimbergo | | E | | Apr | | 2016 | | 3 | | 26 | | 1 | | 2 | |
| LP75_2016_7361 | | LP75 | | Trieste | | S | | May | | 2016 | | 1 | | 19 | | 5 | | 3 | |
| LP76_2016_7363 | | LP76 | | Trieste | | S | | May | | 2016 | | 1 | | 19 | | 3 | | 1 | |
| LP77_2016_8723 | | LP77 | | Udine | | W | | Jun | | 2016 | | 8 | | 16 | | 7 | | 1 | |
| LP78_2016_9232 | | LP78 | | Udine | | W | | Jun | | 2016 | | 1 | | 1 | | 3 | | 1 | |
| LP79_2016_9249 | | LP79 | | Trieste | | S | | Jun | | 2016 | | 1 | | 13 | | 3 | | 3 | |
| LP80_2016_9485 | | LP80 | | Udine | | W | | Jun | | 2016 | | 8 | | 34 | | 7 | | 2 | |
| LP81_2016_9487 | | LP81 | | Udine | | W | | Jun | | 2016 | | 2 | | 15 | | 5 | | 3 | |
| LP83_2016_11187 | | LP83 | | Farra d'Isonzo | | S | | Jul | | 2016 | | 1 | | 16 | | 7 | | 1 | |
| LP84_2016_11195 | | LP84 | | San Pietro al Natisone | | W | | Jul | | 2016 | | 5 | | 44 | | 20 | | 1 | |
| LP85_2016_11282 | | LP85 | | Trieste | | S | | Aug | | 2016 | | 1 | | 13 | | 5 | | 1 | |
| LP86_2016_11325A | | LP86 | | Grado | | S | | Aug | | 2016 | | 1 | | 45 | | 17 | | 1 | |
| LP87_2016_11325B | | LP87 | | Grado | | S | | Aug | | 2016 | | 1 | | 45 | | 17 | | 1 | |
| LP88_2016_11484 | | LP88 | | Monfalcone | | S | | Aug | | 2016 | | 1 | | 28 | | 18 | | 2 | |
| LP89_2016_11555 | | LP89 | | Trieste | | S | | Aug | | 2016 | | 1 | | 22 | | 19 | | 1 | |
| LP90_2016_12593 | | LP90 | | Porcia | | E | | Aug | | 2016 | | 2 | | 26 | | 17 | | 3 | |
| LP91_2016_12594 | | LP91 | | Porcia | | E | | Aug | | 2016 | | 2 | | 26 | | 17 | | 1 | |
| LP92_2016_12602 | | LP92 | | Porcia | | E | | Aug | | 2016 | | 2 | | 26 | | 17 | | 1 | |
| LP93_2016_12604 | | LP93 | | Porcia | | E | | Aug | | 2016 | | 2 | | 26 | | 17 | | 1 | |
| LP94_2016_12605 | | LP94 | | Porcia | | E | | Aug | | 2016 | | 2 | | 42 | | 17 | | 3 | |
| LP95_2016_12642 | | LP95 | | Grado | | S | | Aug | | 2016 | | 1 | | 18 | | 17 | | 3 | |
| LP96_2016_12645 | | LP96 | | Grado | | S | | Aug | | 2016 | | 1 | | 18 | | 1 | | 3 | |
| LP97_2016_12647 | | LP97 | | Grado | | S | | Aug | | 2016 | | 1 | | 19 | | 10 | | 3 | |
| LP98_2016_12649 | | LP98 | | Grado | | s | | Aug | | 2016 | | 1 | | 46 | | 10 | | 2 | |
| LP99_2016_14554 | | LP99 | | Gorizia | | S | | Sep | | 2016 | | 2 | | 23 | | 3 | | 1 | |
| LP103_2016_18273A | | LP103 | | Porpetto | | S | | Dic | | 2016 | | 1 | | 24 | | 10 | | 2 | |
| LP104_2016_18273B | | LP104 | | Porpetto | | S | | Dic | | 2016 | | 1 | | 24 | | 10 | | 1 | |
| LP105_2016_18274A | | LP105 | | Porpetto | | S | | Dic | | 2016 | | 1 | | 24 | | 10 | | 2 | |
| LP106_2016_18274B | | LP106 | | Porpetto | | S | | Dic | | 2016 | | 1 | | 24 | | 10 | | 2 | |
| LP107_2017_92 | | LP107 | | San Quirino | | E | | Jan | | 2017 | | 1 | | 9 | | 3 | | 1 | |
| LP108_2017_94 | | LP108 | | San Quirino | | E | | Jan | | 2017 | | 1 | | 17 | | 3 | | 1 | |
| LP109_2017_96 | | LP109 | | San Quirino | | E | | Jan | | 2017 | | 1 | | 17 | | 7 | | 1 | |
| LP110_2017_100 | | LP110 | | San Quirino | | E | | Jan | | 2017 | | 1 | | 17 | | 3 | | 1 | |
| LP111_2017_2915_A1 | | LP111 | | Spilimbergo | | E | | Feb | | 2017 | | 1 | | 10 | | 3 | | 1 | |
| LP112_2017_2921_A1 | | LP112 | | Spilimbergo | | E | | Feb | | 2017 | | 1 | | 10 | | 3 | | 2 | |
| LP113_2017_3830 | | LP113 | | Lignano Sabbiadoro | | S | | Mar | | 2017 | | 2-15 | | 38 | | 9 | | 1 | |
| LP114_2017_4027 | | LP114 | | Aviano | | W | | Mar | | 2017 | | 1 | | 8 | | 1 | | 3 | |
| LP116_2017_10304 | | LP116 | | Grado | | s | | Jun | | 2017 | | 2-15 | | 13 | | 2 | | 1 | |
| LP118_2017_13076 | | LP118 | | Basiliano | | W | | Aug | | 2017 | | 1 | | 8 | | 1 | | 2 | |
| LP119_2017_13083 | | LP119 | | Basiliano | | W | | Aug | | 2017 | | 1 | | 8 | | 1 | | 2 | |
| LP120_2017_13904 | | LP120 | | Grado | | S | | Aug | | 2017 | | 2-15 | | 17 | | 7 | | 1 | |
| LP121_2017_13917 | | LP121 | | Grado | | S | | Aug | | 2017 | | 2-15 | | 16 | | 7 | | 1 | |
| LP122_2017_14194 | | LP122 | | Aviano | | w | | Aug | | 2017 | | 2-15 | | 46 | | 10 | | 2 | |
| LP123_2017_14198 | | LP123 | | Aviano | | w | | Aug | | 2017 | | 2-15 | | 35 | | 14 | | 2 | |
| LP124_2017_14204 | | LP124 | | Pordenone | | E | | Aug | | 2017 | | 1 | | 47 | | 10 | | 3 | |
| LP125_2017_14205 | | LP125 | | Pordenone | | E | | Aug | | 2017 | | 1 | | 47 | | 10 | | 2 | |
| LP126_2017_14977 | | LP126 | | Gorizia | | S | | Sep | | 2017 | | 1 | | 11 | | 5 | | 3 | |
| LP127_2017_14984 | | LP127 | | Venzone | | W | | Sep | | 2017 | | 2-15 | | 12 | | 5 | | 2 | |
| LP128_2017_15006 | | LP128 | | Venzone | | W | | Sep | | 2017 | | 1 | | 38 | | 7 | | 1 | |
| LP129_2017_15640 | | LP129 | | Bagnaria Arsa | | S | | Sep | | 2017 | | 1 | | 13 | | 1 | | 3 | |
| LP130_2017_15642 | | LP130 | | Bagnaria Arsa | | S | | Sep | | 2017 | | 1 | | 11 | | 4 | | 1 | |
| LP132_2017_15856 | | LP132 | | Palmanova | | S | | Sep | | 2017 | | 1 | | 19 | | 16 | | 2 | |
| LP133_2017_15858 | | LP133 | | Palmanova | | S | | Sep | | 2017 | | 2 | | 15 | | 12 | | 2 | |
| LP134_2017_16867 | | LP134 | | Fontanafredda | | E | | Sep | | 2017 | | 1 | | 13 | | 16 | | 1 | |
| LP137_2017_17239 | | LP137 | | Zoppola | | E | | Oct | | 2017 | | 2 | | 13 | | 3 | | 3 | |
| LP138_2017_17845 | | LP138 | | Udine | | W | | Oct | | 2017 | | 1 | | 11 | | 3 | | 3 | |
| LP140_2017_19555 | | LP140 | | Campoformido | | W | | Mar | | 2017 | | 1 | | 13 | | 19 | | 1 | |
| LP141_2017_19559 | | LP141 | | Campoformido | | W | | Apr | | 2017 | | 1 | | 13 | | 3 | | 3 | |
| LP144_2017_20670 | | LP144 | | Campoformido | | W | | May | | 2017 | | 1 | | 27 | | 12 | | 3 | |
