# Supplementary material for: Genotypic and phenotypic profiling of 127 Legionella pneumophila strains: Insights into regional spread
Source: PLoS One. 2024 Jul 19;19(7):e0307646. doi: 10.1371/journal.pone.0307646 (PMC11259292; doi:10.1371/journal.pone.0307646)
Supplement: S2 Table — (DOCX) [file pone.0307646.s002.docx]

**Table S2.** Fatty acids composition of the different isolated *Lp* strains.

| **Species** | **Strain** | **Fatty Acids percentages (%)** | | | | | | | | | | | | | | | | | | | | | | | | | | | | | | | | | | | | | |
| --- | --- | --- | --- | --- | --- | --- | --- | --- | --- | --- | --- | --- | --- | --- | --- | --- | --- | --- | --- | --- | --- | --- | --- | --- | --- | --- | --- | --- | --- | --- | --- | --- | --- | --- | --- | --- | --- | --- | --- |
|  |  | **i-C14:0** | | **C14:1 cis-9** | **C14:0** | | **i-C15:0** | | **a-C15:0** | | **C15:1 cis-9** | **C15:0** | | | **3-OH-i-C14:0** | **i-C16:1** | | **i-C16:0** | | **C16:1 cis-9** | | **C16:0** | | **i-C17:1** | | **i-C17:0** | | **a-C17:0** | | **C17:0-9 cp** | | **C17:0** | | **i-C18:0** | | **C18:0** | | **C19:0** | **C20:0** |
| *L. pneumophila* | LP1_2005_3688 | 5,55 | | 0,43 | 0,82 | | 0,57 | | 10,70 | | 2,72 | 1,82 | | | 0,46 | 2,27 | | 33,86 | | 14,19 | | 11,67 | | 0,26 | | 0,26 | | 4,67 | | 4,80 | | 1,57 | | 0,29 | | 1,70 | | 0,53 | 0,87 |
| *L. pneumophila* | LP2_2010_7035 | 5,42 | | 0,86 | 0,89 | | 0,58 | | 10,96 | | 3,48 | 1,51 | | | 1,23 | 3,35 | | 24,50 | | 25,52 | | 9,30 | | 0,58 | | 0,55 | | 4,96 | | 2,15 | | 1,26 | | 0,00 | | 1,63 | | 0,40 | 0,86 |
| *L. pneumophila* | LP3_2010_7343 | 6,04 | | 0,38 | 0,46 | | 0,50 | | 11,46 | | 1,99 | 0,73 | | | 0,65 | 4,24 | | 30,98 | | 10,91 | | 6,57 | | 0,74 | | 0,34 | | 5,18 | | 8,71 | | 2,14 | | 0,44 | | 2,66 | | 0,88 | 2,50 |
| *L. pneumophila* | LP4_2010_12257 | 6,57 | | 0,53 | 0,85 | | 0,69 | | 9,63 | | 2,45 | 1,04 | | | 0,67 | 2,66 | | 29,24 | | 12,93 | | 10,14 | | 0,00 | | 0,35 | | 4,34 | | 8,75 | | 2,02 | | 0,00 | | 2,71 | | 0,74 | 1,76 |
| *L. pneumophila* | LP5_2010_12508 | 7,65 | | 0,93 | 0,87 | | 0,60 | | 12,26 | | 4,00 | 1,51 | | | 0,80 | 3,42 | | 26,40 | | 15,23 | | 8,09 | | 0,44 | | 0,36 | | 3,52 | | 8,93 | | 1,64 | | 0,00 | | 1,80 | | 0,44 | 1,10 |
| *L. pneumophila* | LP6_2010_13100 | 6,21 | | 0,55 | 0,97 | | 0,47 | | 10,40 | | 3,01 | 1,85 | | | 0,99 | 3,20 | | 23,29 | | 12,56 | | 9,24 | | 0,00 | | 3,53 | | 0,99 | | 8,89 | | 2,35 | | 0,00 | | 2,54 | | 1,08 | 2,40 |
| *L. pneumophila* | LP7_2010_13335 | 5,73 | | 0,41 | 0,59 | | 0,79 | | 11,69 | | 2,26 | 1,45 | | | 0,67 | 2,06 | | 34,29 | | 11,81 | | 9,27 | | 0,29 | | 0,28 | | 6,25 | | 5,04 | | 1,59 | | 0,31 | | 3,01 | | 0,69 | 1,54 |
| *L. pneumophila* | LP8_2010_13575 | 7,22 | | 0,99 | 0,84 | | 0,45 | | 12,46 | | 4,06 | 1,34 | | | 0,86 | 3,30 | | 27,21 | | 22,40 | | 7,34 | | 0,49 | | 0,21 | | 4,20 | | 3,71 | | 0,78 | | 0,09 | | 1,08 | | 0,41 | 0,53 |
| *L. pneumophila* | LP9_2010_13639 | 8,99 | | 0,93 | 0,65 | | 0,65 | | 13,21 | | 3,94 | 1,48 | | | 1,30 | 4,13 | | 30,60 | | 13,58 | | 5,05 | | 0,00 | | 0,00 | | 4,13 | | 6,63 | | 1,67 | | 0,00 | | 1,81 | | 0,00 | 1,25 |
| *L. pneumophila* | LP10_2010_14262 | 4,85 | | 0,64 | 0,59 | | 0,40 | | 9,40 | | 3,00 | 1,17 | | | 0,57 | 2,24 | | 28,93 | | 20,25 | | 8,99 | | 0,31 | | 0,36 | | 5,90 | | 2,86 | | 1,78 | | 0,00 | | 2,55 | | 1,12 | 2,59 |
| *L. pneumophila* | LP11_2010_14388 | 6,10 | | 0,62 | 0,51 | | 0,52 | | 10,85 | | 3,14 | 1,26 | | | 0,83 | 3,24 | | 31,53 | | 13,30 | | 7,96 | | 0,52 | | 0,56 | | 5,60 | | 3,95 | | 2,12 | | 0,10 | | 2,82 | | 1,35 | 3,10 |
| *L. pneumophila* | LP12_2010_14946 | 4,70 | | 0,68 | 0,61 | | 0,35 | | 9,07 | | 2,99 | 1,28 | | | 0,67 | 2,13 | | 29,30 | | 22,62 | | 9,44 | | 0,27 | | 0,35 | | 5,99 | | 0,99 | | 1,59 | | 0,00 | | 2,86 | | 1,21 | 2,88 |
| *L. pneumophila* | LP13_2010_15075 | 5,36 | | 0,82 | 1,20 | | 0,00 | | 11,36 | | 3,60 | 2,33 | | | 0,63 | 2,33 | | 26,18 | | 17,35 | | 11,04 | | 0,00 | | 0,00 | | 4,67 | | 5,55 | | 2,15 | | 0,00 | | 2,33 | | 0,95 | 2,15 |
| *L. pneumophila* | LP14_2011_1360 | 9,35 | | 1,10 | 0,66 | | 0,61 | | 19,44 | | 3,35 | 1,07 | | | 1,21 | 5,19 | | 28,41 | | 10,96 | | 5,51 | | 1,10 | | 0,00 | | 4,53 | | 5,02 | | 0,61 | | 0,00 | | 1,21 | | 0,00 | 0,69 |
| *L. pneumophila* | LP15_2011_2680 | 5,58 | | 0,67 | 0,70 | | 0,51 | | 16,16 | | 2,51 | 1,29 | | | 0,65 | 3,40 | | 27,88 | | 9,24 | | 8,31 | | 1,18 | | 0,26 | | 6,32 | | 9,10 | | 1,67 | | 0,21 | | 2,05 | | 0,81 | 1,49 |
| *L. pneumophila* | LP16_2011_2730 | 6,24 | | 0,42 | 0,67 | | 0,76 | | 9,93 | | 2,17 | 1,89 | | | 0,57 | 2,37 | | 36,94 | | 11,15 | | 9,60 | | 0,38 | | 0,30 | | 4,98 | | 5,38 | | 1,81 | | 0,29 | | 2,05 | | 0,74 | 1,35 |
| *L. pneumophila* | LP17_2011_4965 | 5,07 | | 0,48 | 0,50 | | 0,43 | | 10,18 | | 2,53 | 1,00 | | | 0,59 | 2,04 | | 31,92 | | 12,58 | | 8,33 | | 0,36 | | 0,32 | | 6,49 | | 6,24 | | 2,01 | | 0,36 | | 2,78 | | 1,79 | 2,33 |
| *L. pneumophila* | LP18_2011_5016 | 3,86 | | 0,50 | 0,63 | | 0,38 | | 8,99 | | 2,45 | 1,36 | | | 0,50 | 1,70 | | 29,33 | | 16,50 | | 10,57 | | 0,44 | | 0,34 | | 6,77 | | 5,37 | | 2,54 | | 0,34 | | 3,25 | | 1,17 | 2,26 |
| *L. pneumophila* | LP19_2011_5017 | 7,75 | | 0,72 | 0,70 | | 0,58 | | 12,73 | | 4,16 | 1,15 | | | 0,87 | 3,39 | | 29,72 | | 13,98 | | 7,10 | | 0,31 | | 0,31 | | 4,36 | | 5,41 | | 1,66 | | 0,00 | | 1,71 | | 0,51 | 1,32 |
| *L. pneumophila* | LP20_2011_5054 | 6,44 | | 0,52 | 0,84 | | 0,52 | | 10,99 | | 2,71 | 1,69 | | | 0,33 | 2,43 | | 33,89 | | 14,85 | | 10,91 | | 0,25 | | 0,22 | | 3,91 | | 5,11 | | 1,21 | | 0,23 | | 1,42 | | 0,44 | 0,70 |
| *L. pneumophila* | LP21_2011_6872 | 6,73 | | 0,43 | 0,52 | | 0,55 | | 10,66 | | 3,18 | 1,19 | | | 0,62 | 3,11 | | 38,90 | | 11,48 | | 7,66 | | 0,28 | | 0,28 | | 4,57 | | 4,47 | | 1,44 | | 0,30 | | 1,67 | | 0,78 | 1,22 |
| *L. pneumophila* | LP22_2011_7143 | 5,52 | | 0,79 | 0,93 | | 0,64 | | 11,03 | | 3,72 | 2,26 | | | 0,75 | 3,22 | | 24,86 | | 14,51 | | 10,21 | | 0,57 | | 0,21 | | 4,58 | | 7,66 | | 2,65 | | 0,00 | | 2,58 | | 0,93 | 2,36 |
| *L. pneumophila* | LP23_2011_7324 | 11,11 | | 0,89 | 0,47 | | 0,70 | | 14,62 | | 2,44 | 0,41 | | | 2,22 | 5,41 | | 33,30 | | 11,46 | | 4,21 | | 0,51 | | 0,16 | | 3,80 | | 4,15 | | 0,51 | | 0,00 | | 0,51 | | 0,00 | 0,82 |
| *L. pneumophila* | LP24_2011_7512 | 7,39 | | 0,60 | 0,71 | | 0,54 | | 10,90 | | 3,04 | 1,13 | | | 0,66 | 4,71 | | 32,70 | | 11,20 | | 6,37 | | 0,00 | | 0,00 | | 4,76 | | 9,17 | | 1,97 | | 0,00 | | 2,44 | | 0,00 | 1,73 |
| *L. pneumophila* | LP25_2011_7660 | 10,61 | | 0,98 | 0,65 | | 0,75 | | 11,91 | | 4,45 | 1,20 | | | 0,93 | 3,54 | | 29,12 | | 14,05 | | 6,98 | | 0,30 | | 0,35 | | 2,93 | | 7,35 | | 1,23 | | 0,17 | | 1,39 | | 0,47 | 0,61 |
| *L. pneumophila* | LP26_2011_7708 | 7,61 | 0,55 | | | 0,74 | 0,72 | 11,67 | | 3,38 | | | 1,00 | 0,55 | | | 3,10 | | 29,24 | | 14,11 | | 7,63 | | 0,28 | | 0,28 | | 4,21 | | 7,04 | | 1,81 | | 0,00 | | 1,79 | 0,57 | 1,13 |
| *L. pneumophila* | LP27_2011_7798 | 6,56 | 0,52 | | | 0,74 | 0,55 | 10,11 | | 2,79 | | | 1,17 | 0,74 | | | 2,70 | | 29,78 | | 13,91 | | 8,74 | | 0,00 | | 0,00 | | 4,70 | | 9,26 | | 2,24 | | 0,00 | | 2,87 | 0,71 | 1,91 |
| *L. pneumophila* | LP28_2011_9951 | 8,31 | 0,55 | | | 0,34 | 0,60 | 14,06 | | 2,10 | | | 0,52 | 1,55 | | | 4,52 | | 36,44 | | 11,91 | | 4,57 | | 0,54 | | 0,34 | | 5,82 | | 3,68 | | 0,67 | | 0,23 | | 1,38 | 0,57 | 1,29 |
| *L. pneumophila* | LP30_2015_4925 | 5,80 | 0,29 | | | 0,77 | 0,37 | 8,01 | | 2,21 | | | 2,13 | 0,37 | | | 0,92 | | 41,61 | | 12,31 | | 12,04 | | 0,08 | | 0,23 | | 4,30 | | 2,54 | | 1,60 | | 0,34 | | 1,96 | 0,70 | 1,23 |
| *L. pneumophila* | LP31_2015_4961 | 4,01 | 0,25 | | | 0,85 | 0,33 | 8,01 | | 1,94 | | | 2,12 | 0,40 | | | 0,76 | | 35,38 | | 13,42 | | 16,41 | | 0,10 | | 0,27 | | 5,23 | | 2,68 | | 1,82 | | 0,31 | | 2,72 | 0,92 | 1,96 |
| *L. pneumophila* | LP32_2015_6923 | 4,57 | 0,00 | | | 0,96 | 0,34 | 8,93 | | 1,74 | | | 1,52 | 0,59 | | | 3,20 | | 26,14 | | 12,91 | | 14,56 | | 0,00 | | 0,00 | | 6,10 | | 3,76 | | 1,93 | | 0,34 | | 4,54 | 1,09 | 3,45 |
| *L. pneumophila* | LP33_2015_6924 | 5,66 | 0,52 | | | 0,73 | 0,47 | 11,64 | | 2,70 | | | 1,27 | 0,49 | | | 2,44 | | 31,68 | | 15,65 | | 10,66 | | 0,28 | | 0,24 | | 5,35 | | 3,84 | | 1,45 | | 0,28 | | 2,29 | 0,72 | 1,65 |
| *L. pneumophila* | LP34_2015_6925 | 4,39 | 0,46 | | | 0,72 | 0,49 | 9,55 | | 2,38 | | | 1,36 | 0,46 | | | 2,49 | | 29,38 | | 16,18 | | 12,10 | | 0,40 | | 0,34 | | 5,92 | | 4,69 | | 2,40 | | 0,34 | | 3,19 | 0,90 | 1,84 |
| *L. pneumophila* | LP38_2015_9504 | 4,91 | 0,35 | | | 0,87 | 0,39 | 9,57 | | 2,35 | | | 1,87 | 0,50 | | | 1,23 | | 31,91 | | 18,35 | | 13,04 | | 0,15 | | 0,27 | | 5,20 | | 0,86 | | 1,55 | | 0,30 | | 2,41 | 1,06 | 2,83 |
| *L. pneumophila* | LP39_2015_9507 | 5,29 | 0,41 | | | 0,75 | 0,41 | 10,26 | | 2,56 | | | 1,74 | 0,40 | | | 1,54 | | 34,10 | | 18,21 | | 10,71 | | 0,18 | | 0,28 | | 5,17 | | 1,13 | | 1,54 | | 0,28 | | 1,93 | 0,90 | 2,16 |
| *L. pneumophila* | LP40_2015_11509 | 3,03 | 0,32 | | | 1,45 | 0,34 | 7,92 | | 1,41 | | | 1,84 | 0,43 | | | 0,65 | | 22,14 | | 19,46 | | 23,78 | | 0,11 | | 0,29 | | 5,73 | | 1,47 | | 1,52 | | 0,27 | | 4,33 | 0,86 | 2,55 |
| *L. pneumophila* | LP41_2015_11525 | 4,39 | 0,27 | | | 0,79 | 0,34 | 10,22 | | 1,99 | | | 1,91 | 0,34 | | | 0,98 | | 34,51 | | 14,86 | | 14,03 | | 0,15 | | 0,27 | | 6,03 | | 2,65 | | 1,56 | | 0,26 | | 2,22 | 0,70 | 1,45 |
| *L. pneumophila* | LP42_2015_11526 | 3,98 | 0,25 | | | 0,97 | 0,33 | 9,62 | | 2,34 | | | 2,12 | 0,46 | | | 0,68 | | 30,99 | | 17,21 | | 16,95 | | 0,08 | | 0,19 | | 5,44 | | 2,43 | | 1,59 | | 0,22 | | 2,21 | 0,59 | 1,27 |
| *L. pneumophila* | LP43_2015_11527 | 3,80 | 0,29 | | | 0,96 | 0,38 | 8,13 | | 1,70 | | | 1,85 | 0,36 | | | 0,91 | | 29,94 | | 15,10 | | 16,94 | | 0,16 | | 0,35 | | 6,13 | | 2,79 | | 1,95 | | 0,36 | | 3,61 | 1,13 | 2,89 |
| *L. pneumophila* | LP44_2015_11755 | 7,90 | 0,79 | | | 0,79 | 0,60 | 13,87 | | 4,43 | | | 1,40 | 0,73 | | | 3,14 | | 27,69 | | 15,92 | | 6,92 | | 0,40 | | 0,30 | | 4,06 | | 6,94 | | 1,51 | | 0,12 | | 1,35 | 0,38 | 0,76 |
| *L. pneumophila* | LP45_2015_11756 | 8,38 | 0,76 | | | 0,51 | 0,45 | 11,55 | | 4,88 | | | 1,06 | 1,09 | | | 2,89 | | 32,60 | | 17,19 | | 5,14 | | 0,35 | | 0,20 | | 4,05 | | 4,29 | | 1,47 | | 0,18 | | 1,22 | 0,67 | 0,87 |
| *L. pneumophila* | LP46_2015_11762 | 7,92 | 0,70 | | | 0,88 | 0,67 | 13,25 | | 4,31 | | | 1,44 | 1,06 | | | 3,34 | | 26,68 | | 16,77 | | 7,45 | | 0,41 | | 0,29 | | 4,02 | | 6,45 | | 1,70 | | 0,00 | | 1,41 | 0,32 | 0,94 |
| *L. pneumophila* | LP47_2015_11763 | 6,72 | 0,27 | | | 0,42 | 0,40 | 9,80 | | 1,66 | | | 0,88 | 0,46 | | | 2,44 | | 47,30 | | 10,08 | | 7,06 | | 0,27 | | 0,24 | | 5,06 | | 3,20 | | 0,65 | | 0,26 | | 1,32 | 0,41 | 1,03 |
| *L. pneumophila* | LP48_2015_11765 | 7,40 | 0,30 | | | 0,53 | 0,49 | 10,36 | | 2,14 | | | 1,18 | 0,61 | | | 2,34 | | 44,50 | | 10,24 | | 7,11 | | 0,26 | | 0,24 | | 4,84 | | 4,07 | | 0,85 | | 0,24 | | 1,16 | 0,37 | 0,77 |
| *L. pneumophila* | LP49_2015_12212 | 4,40 | 0,40 | | | 0,55 | 0,42 | 8,40 | | 1,80 | | | 1,59 | 0,54 | | | 1,99 | | 31,35 | | 13,05 | | 12,37 | | 0,21 | | 0,26 | | 5,86 | | 6,98 | | 2,31 | | 0,46 | | 3,71 | 0,94 | 2,40 |
| *L. pneumophila* | LP50_2015_12213 | 12,37 | 0,69 | | | 0,34 | 0,54 | 11,64 | | 4,65 | | | 0,79 | 1,04 | | | 4,71 | | 38,68 | | 11,70 | | 2,97 | | 0,33 | | 0,25 | | 2,82 | | 3,52 | | 0,87 | | 0,21 | | 0,85 | 0,47 | 0,54 |
| *L. pneumophila* | LP51_2015_12754 | 10,28 | 0,83 | | | 1,09 | 0,64 | 12,60 | | 3,96 | | | 1,26 | 1,34 | | | 3,40 | | 27,26 | | 16,13 | | 6,66 | | 0,28 | | 0,21 | | 3,13 | | 6,92 | | 1,30 | | 0,00 | | 1,19 | 0,30 | 0,85 |
| *L. pneumophila* | LP52_2015_12755 | 7,82 | 0,82 | | | 0,70 | 0,50 | 13,13 | | 4,52 | | | 1,78 | 1,16 | | | 3,00 | | 29,06 | | 15,52 | | 6,62 | | 0,21 | | 0,20 | | 4,25 | | 4,44 | | 1,29 | | 0,00 | | 1,36 | 0,37 | 0,66 |
| *L. pneumophila* | LP53_2015_12756 | 10,70 | 1,28 | | | 1,09 | 0,55 | 14,42 | | 2,80 | | | 0,86 | 1,69 | | | 5,32 | | 23,98 | | 17,65 | | 7,16 | | 0,72 | | 0,12 | | 2,83 | | 5,46 | | 0,61 | | 0,12 | | 1,32 | 0,35 | 1,00 |
| *L. pneumophila* | LP54_2015_12757 | 6,54 | 0,00 | | | 0,71 | 0,48 | 11,27 | | 1,28 | | | 0,83 | 0,84 | | | 3,92 | | 30,98 | | 10,51 | | 10,57 | | 0,00 | | 0,29 | | 5,03 | | 5,61 | | 0,88 | | 0,40 | | 3,59 | 0,87 | 3,90 |
| *L. pneumophila* | LP56_2015_13149 | 8,35 | 1,25 | | | 1,08 | 0,53 | 14,59 | | 2,56 | | | 0,79 | 1,35 | | | 4,30 | | 21,79 | | 18,86 | | 8,35 | | 0,66 | | 0,00 | | 3,84 | | 6,87 | | 0,76 | | 0,00 | | 2,17 | 0,00 | 1,12 |
| *L. pneumophila* | LP57_2015_13150 | 8,35 | 1,09 | | | 0,84 | 0,54 | 13,98 | | 2,17 | | | 0,64 | 1,48 | | | 5,78 | | 24,79 | | 18,32 | | 7,01 | | 0,84 | | 0,00 | | 3,70 | | 6,57 | | 0,64 | | 0,00 | | 1,83 | 0,00 | 1,43 |
| *L. pneumophila* | LP58_2015_13151 | 5,80 | 0,46 | | | 0,70 | 0,46 | 11,89 | | 1,46 | | | 0,88 | 0,45 | | | 3,71 | | 31,77 | | 13,19 | | 11,48 | | 0,65 | | 0,21 | | 5,28 | | 5,30 | | 0,80 | | 0,27 | | 2,55 | 0,55 | 2,13 |
| *L. pneumophila* | LP59_2015_13152 | 7,91 | 0,00 | | | 0,83 | 0,42 | 13,10 | | 1,65 | | | 1,00 | 0,83 | | | 4,80 | | 29,05 | | 12,45 | | 10,31 | | 0,78 | | 0,13 | | 4,26 | | 5,82 | | 1,13 | | 0,20 | | 2,19 | 0,34 | 1,03 |
| *L. pneumophila* | LP60_2015_13153 | 9,26 | 1,00 | | | 1,39 | 0,46 | 16,36 | | 3,20 | | | 1,27 | 1,38 | | | 3,87 | | 23,35 | | 17,06 | | 7,74 | | 0,67 | | 0,00 | | 3,22 | | 6,56 | | 0,50 | | 0,00 | | 1,35 | 0,36 | 1,01 |
| *L. pneumophila* | LP61_2015_13154 | 6,32 | 0,44 | | | 0,82 | 0,50 | 11,50 | | 2,90 | | | 1,57 | 0,40 | | | 2,56 | | 32,25 | | 16,13 | | 10,72 | | 0,27 | | 0,21 | | 3,99 | | 5,22 | | 1,31 | | 0,20 | | 1,39 | 0,45 | 0,85 |
| *L. pneumophila* | LP62_2015_13155 | 9,19 | 1,03 | | | 0,82 | 0,54 | 12,39 | | 4,69 | | | 1,32 | 1,41 | | | 3,97 | | 26,81 | | 16,83 | | 6,75 | | 0,42 | | 0,20 | | 3,36 | | 6,09 | | 1,42 | | 0,18 | | 1,22 | 0,59 | 0,77 |
| *L. pneumophila* | LP63_2016_1544 | 5,11 | 0,41 | | | 0,60 | 0,40 | 9,44 | | 2,38 | | | 1,36 | 0,64 | | | 2,81 | | 30,88 | | 13,47 | | 10,40 | | 0,35 | | 0,27 | | 5,79 | | 4,66 | | 2,41 | | 0,40 | | 4,03 | 1,17 | 3,02 |
| *L. pneumophila* | LP64_2016_1756 | 5,18 | 0,98 | | | 1,01 | 0,47 | 10,82 | | 3,26 | | | 1,27 | 0,93 | | | 2,43 | | 26,74 | | 23,09 | | 9,32 | | 0,36 | | 0,31 | | 5,33 | | 2,87 | | 1,48 | | 0,00 | | 2,12 | 0,60 | 1,42 |
| *L. pneumophila* | LP65_2016_1763 | 4,50 | 0,69 | | | 0,83 | 0,40 | 9,75 | | 2,64 | | | 1,43 | 0,79 | | | 2,15 | | 27,65 | | 21,68 | | 11,00 | | 0,35 | | 0,31 | | 5,87 | | 2,44 | | 1,43 | | 0,26 | | 2,77 | 0,82 | 2,23 |
| *L. pneumophila* | LP66_2016_1764 | 6,77 | 0,41 | | | 0,73 | 0,98 | 10,92 | | 2,28 | | | 1,80 | 0,81 | | | 2,45 | | 31,23 | | 12,57 | | 10,44 | | 0,51 | | 0,24 | | 4,59 | | 6,45 | | 1,75 | | 0,18 | | 2,43 | 0,68 | 1,77 |
| *L. pneumophila* | LP67_2016_1798 | 4,74 | 0,30 | | | 0,56 | 0,37 | 10,69 | | 2,90 | | | 1,10 | 0,97 | | | 2,51 | | 29,83 | | 17,09 | | 11,19 | | 0,36 | | 0,30 | | 5,37 | | 1,31 | | 1,71 | | 0,54 | | 4,16 | 1,27 | 2,73 |
| *L. pneumophila* | LP68_2016_1801 | 8,17 | 1,02 | | | 1,02 | 0,39 | 11,94 | | 4,86 | | | 1,62 | 1,02 | | | 3,13 | | 26,98 | | 22,68 | | 7,54 | | 0,00 | | 0,00 | | 3,56 | | 3,49 | | 0,85 | | 0,00 | | 1,13 | 0,00 | 0,60 |
| *L. pneumophila* | LP69_2016_2458 | 6,33 | 0,50 | | | 0,56 | 0,50 | 11,02 | | 2,12 | | | 1,13 | 0,45 | | | 3,13 | | 34,86 | | 12,19 | | 8,73 | | 0,36 | | 0,30 | | 5,48 | | 6,14 | | 1,39 | | 0,29 | | 1,96 | 0,67 | 1,50 |
| *L. pneumophila* | LP70_2016_2460 | 4,84 | 0,43 | | | 0,84 | 0,52 | 10,06 | | 2,03 | | | 1,63 | 0,46 | | | 2,19 | | 30,96 | | 14,40 | | 14,78 | | 0,32 | | 0,34 | | 5,81 | | 3,82 | | 1,38 | | 0,34 | | 2,38 | 0,70 | 1,46 |
| *L. pneumophila* | LP71_2016_4320 | 4,80 | 0,36 | | | 1,03 | 0,55 | 8,61 | | 2,25 | | | 2,27 | 0,73 | | | 1,97 | | 29,62 | | 16,84 | | 13,88 | | 0,16 | | 0,22 | | 4,52 | | 3,52 | | 2,23 | | 0,39 | | 3,38 | 0,95 | 1,72 |
| *L. pneumophila* | LP72_2016_4381 | 4,12 | 0,46 | | | 0,87 | 0,39 | 8,44 | | 2,15 | | | 2,23 | 0,59 | | | 1,69 | | 27,13 | | 16,42 | | 14,12 | | 0,23 | | 0,27 | | 5,13 | | 3,85 | | 3,08 | | 0,37 | | 3,96 | 1,30 | 3,19 |
| *L. pneumophila* | LP73_2016_4407 | 5,10 | 0,85 | | | 1,02 | 0,51 | 11,97 | | 3,18 | | | 1,70 | 0,51 | | | 2,72 | | 27,17 | | 21,27 | | 10,89 | | 0,00 | | 0,00 | | 6,07 | | 2,38 | | 1,42 | | 0,00 | | 2,04 | 0,00 | 1,19 |
| *L. pneumophila* | LP74_2016_4429 | 3,51 | 0,41 | | | 0,94 | 0,61 | 8,43 | | 1,80 | | | 2,06 | 0,74 | | | 1,84 | | 27,20 | | 15,96 | | 15,88 | | 0,00 | | 0,00 | | 5,47 | | 3,92 | | 2,63 | | 0,39 | | 4,27 | 1,12 | 2,80 |
| *L. pneumophila* | LP75_2016_7361 | 6,48 | 0,54 | | | 1,28 | 0,42 | 9,99 | | 2,88 | | | 2,15 | 0,80 | | | 2,39 | | 28,28 | | 19,94 | | 12,71 | | 0,21 | | 0,14 | | 3,43 | | 3,52 | | 1,55 | | 0,23 | | 1,87 | 0,45 | 0,73 |
| *L. pneumophila* | LP76_2016_7363 | 4,45 | 0,34 | | | 1,04 | 0,40 | 8,51 | | 1,93 | | | 2,19 | 0,63 | | | 1,85 | | 30,33 | | 18,37 | | 15,50 | | 0,23 | | 0,11 | | 4,67 | | 3,00 | | 1,98 | | 0,28 | | 2,51 | 0,64 | 1,04 |
| *L. pneumophila* | LP77_2016_8723 | 7,06 | 0,71 | | | 1,06 | 0,53 | 11,70 | | 3,12 | | | 1,67 | 0,00 | | | 3,08 | | 29,80 | | 15,75 | | 10,17 | | 0,00 | | 0,00 | | 4,35 | | 6,80 | | 1,51 | | 0,00 | | 1,56 | 0,00 | 1,12 |
| *L. pneumophila* | LP78_2016_9232 | 5,61 | 0,43 | | | 0,94 | 0,53 | 9,96 | | 2,45 | | | 1,88 | 0,40 | | | 2,05 | | 32,93 | | 15,38 | | 14,11 | | 0,20 | | 0,23 | | 4,05 | | 4,29 | | 1,35 | | 0,22 | | 1,63 | 0,49 | 0,78 |
| *L. pneumophila* | LP79_2016_9249 | 8,08 | 0,00 | | | 1,13 | 0,79 | 10,93 | | 2,38 | | | 2,17 | 1,02 | | | 1,74 | | 33,17 | | 11,86 | | 12,94 | | 0,00 | | 0,00 | | 4,05 | | 4,34 | | 1,29 | | 0,00 | | 1,65 | 0,38 | 0,63 |
| *L. pneumophila* | LP80_2016_9485 | 4,46 | 0,62 | | | 0,68 | 0,34 | 8,46 | | 2,19 | | | 0,88 | 0,68 | | | 2,07 | | 28,43 | | 24,79 | | 10,20 | | 0,31 | | 0,37 | | 5,91 | | 1,14 | | 1,33 | | 0,31 | | 3,15 | 0,99 | 2,67 |
| *L. pneumophila* | LP81_2016_9487 | 9,17 | 0,00 | | | 1,10 | 0,69 | 11,86 | | 2,76 | | | 1,60 | 1,76 | | | 2,48 | | 26,46 | | 19,02 | | 11,33 | | 0,00 | | 0,00 | | 4,27 | | 1,29 | | 1,04 | | 0,00 | | 1,38 | 0,00 | 0,85 |
| *L. pneumophila* | LP83_2016_11187 | 5,90 | 0,00 | | | 0,85 | 0,61 | 10,95 | | 1,91 | | | 1,72 | 0,68 | | | 2,26 | | 32,69 | | 10,25 | | 12,53 | | 0,00 | | 0,30 | | 5,53 | | 5,53 | | 1,72 | | 0,35 | | 2,75 | 0,85 | 1,97 |
| *L. pneumophila* | LP84_2016_11195 | 6,43 | 0,53 | | | 0,82 | 0,49 | 12,24 | | 1,82 | | | 1,04 | 0,44 | | | 3,51 | | 31,00 | | 14,73 | | 11,64 | | 0,57 | | 0,17 | | 4,49 | | 4,89 | | 0,66 | | 0,22 | | 2,16 | 0,47 | 1,70 |
| *L. pneumophila* | LP85_2016_11282 | 6,20 | 0,64 | | | 0,61 | 0,48 | 10,16 | | 3,06 | | | 1,13 | 0,77 | | | 2,77 | | 31,14 | | 14,28 | | 9,31 | | 0,39 | | 0,39 | | 4,83 | | 6,28 | | 2,26 | | 0,32 | | 2,43 | 1,01 | 1,56 |
| *L. pneumophila* | LP86_2016_11325A | 5,02 | 0,55 | | | 0,75 | 0,60 | 11,89 | | 2,62 | | | 1,32 | 0,43 | | | 2,09 | | 28,90 | | 18,39 | | 11,59 | | 0,30 | | 0,25 | | 5,11 | | 5,16 | | 1,43 | | 0,21 | | 1,90 | 0,45 | 0,92 |
| *L. pneumophila* | LP87_2016_11325B | 5,10 | 0,00 | | | 0,84 | 0,46 | 9,47 | | 2,28 | | | 1,18 | 1,25 | | | 2,09 | | 25,97 | | 14,64 | | 14,56 | | 0,00 | | 0,00 | | 5,36 | | 4,41 | | 2,13 | | 0,00 | | 3,35 | 0,76 | 1,79 |
| *L. pneumophila* | LP88_2016_11484 | 4,21 | 0,48 | | | 0,87 | 0,50 | 9,00 | | 2,34 | | | 1,89 | 0,84 | | | 1,97 | | 26,65 | | 17,56 | | 13,98 | | 0,28 | | 0,30 | | 5,39 | | 3,96 | | 2,56 | | 0,36 | | 3,53 | 1,07 | 2,28 |
| *L. pneumophila* | LP89_2016_11555 | 5,43 | 0,48 | | | 0,82 | 0,53 | 9,93 | | 2,39 | | | 1,60 | 0,35 | | | 2,24 | | 34,37 | | 13,58 | | 12,16 | | 0,23 | | 0,27 | | 4,96 | | 4,69 | | 1,63 | | 0,33 | | 2,02 | 0,68 | 1,20 |
| *L. pneumophila* | LP90_2016_12593 | 8,49 | 0,90 | | | 0,33 | 0,63 | 12,98 | | 3,20 | | | 0,77 | 1,73 | | | 5,56 | | 36,15 | | 10,39 | | 3,63 | | 0,63 | | 0,33 | | 5,63 | | 4,49 | | 0,93 | | 0,00 | | 1,53 | 0,57 | 1,13 |
| *L. pneumophila* | LP91_2016_12594 | 5,27 | 0,42 | | | 0,80 | 0,53 | 10,82 | | 2,40 | | | 1,48 | 0,88 | | | 2,47 | | 32,56 | | 14,61 | | 12,34 | | 0,33 | | 0,26 | | 5,33 | | 3,86 | | 1,28 | | 0,24 | | 1,86 | 0,65 | 1,38 |
| *L. pneumophila* | LP92_2016_12602 | 5,35 | 0,46 | | | 0,54 | 0,66 | 10,57 | | 3,03 | | | 1,44 | 0,60 | | | 3,08 | | 30,51 | | 13,77 | | 9,05 | | 0,50 | | 0,32 | | 5,54 | | 7,16 | | 2,35 | | 0,29 | | 2,43 | 0,86 | 1,49 |
| *L. pneumophila* | LP93_2016_12604 | 4,45 | 0,51 | | | 0,87 | 0,50 | 9,35 | | 2,36 | | | 1,71 | 0,49 | | | 1,67 | | 31,14 | | 14,76 | | 14,79 | | 0,19 | | 0,27 | | 5,32 | | 3,72 | | 1,86 | | 0,31 | | 2,64 | 0,85 | 2,22 |
| *L. pneumophila* | LP94_2016_12605 | 7,01 | 0,56 | | | 0,64 | 0,53 | 11,35 | | 3,14 | | | 1,09 | 1,49 | | | 2,52 | | 32,85 | | 14,52 | | 7,26 | | 0,25 | | 0,31 | | 4,99 | | 5,66 | | 1,32 | | 0,00 | | 2,07 | 0,67 | 1,77 |
| *L. pneumophila* | LP95_2016_12642 | 5,45 | 0,89 | | | 0,86 | 0,44 | 11,40 | | 4,23 | | | 1,51 | 0,83 | | | 2,58 | | 25,30 | | 23,37 | | 8,50 | | 0,37 | | 0,26 | | 5,29 | | 2,74 | | 1,41 | | 0,00 | | 2,06 | 0,68 | 1,51 |
| *L. pneumophila* | LP96_2016_12645 | 6,54 | 1,31 | | | 1,09 | 0,48 | 12,70 | | 5,13 | | | 1,46 | 1,03 | | | 2,74 | | 21,56 | | 28,70 | | 7,48 | | 0,43 | | 0,17 | | 3,67 | | 2,45 | | 0,91 | | 0,06 | | 1,13 | 0,41 | 0,56 |
| *L. pneumophila* | LP97_2016_12647 | 10,95 | 1,49 | | | 0,56 | 0,54 | 13,66 | | 6,36 | | | 1,10 | 1,95 | | | 4,22 | | 29,23 | | 18,42 | | 3,69 | | 0,45 | | 0,19 | | 3,30 | | 1,71 | | 0,53 | | 0,15 | | 0,79 | 0,33 | 0,38 |
| *L. pneumophila* | LP98_2016_12649 | 4,45 | 0,44 | | | 0,43 | 0,45 | 10,65 | | 2,78 | | | 1,13 | 0,77 | | | 2,97 | | 28,36 | | 15,23 | | 7,24 | | 0,51 | | 0,37 | | 6,32 | | 5,88 | | 2,08 | | 1,91 | | 2,85 | 0,91 | 1,90 |
| *L. pneumophila* | LP99_2016_14554 | 8,42 | 0,34 | | | 0,46 | 0,52 | 7,99 | | 2,23 | | | 1,24 | 0,42 | | | 2,49 | | 49,27 | | 10,50 | | 6,08 | | 0,15 | | 0,18 | | 3,10 | | 3,41 | | 0,85 | | 0,25 | | 1,00 | 0,36 | 0,66 |
| *L. pneumophila* | LP103_2016_18273A | 5,21 | 0,00 | | | 0,79 | 0,54 | 9,87 | | 2,07 | | | 1,49 | 0,61 | | | 2,21 | | 26,43 | | 12,35 | | 14,78 | | 0,00 | | 0,00 | | 5,52 | | 5,00 | | 2,54 | | 0,50 | | 4,03 | 1,06 | 3,58 |
| *L. pneumophila* | LP104_2016_18273B | 4,16 | 0,47 | | | 0,76 | 1,38 | 10,88 | | 2,52 | | | 1,97 | 1,11 | | | 2,45 | | 25,04 | | 15,75 | | 12,37 | | 0,47 | | 0,47 | | 4,77 | | 5,39 | | 2,97 | | 0,00 | | 3,75 | 0,90 | 2,44 |
| *L. pneumophila* | LP105_2016_18274A | 3,94 | 0,48 | | | 1,06 | 0,36 | 8,39 | | 1,99 | | | 2,06 | 0,59 | | | 1,79 | | 26,60 | | 18,02 | | 16,58 | | 0,27 | | 0,18 | | 5,01 | | 2,87 | | 2,38 | | 0,32 | | 3,61 | 1,00 | 2,50 |
| *L. pneumophila* | LP106_2016_18274B | 4,10 | 0,44 | | | 0,97 | 0,37 | 8,36 | | 2,32 | | | 2,39 | 0,66 | | | 1,87 | | 26,01 | | 16,97 | | 15,08 | | 0,23 | | 0,24 | | 5,02 | | 3,22 | | 2,70 | | 0,39 | | 4,17 | 1,21 | 3,26 |
| *L. pneumophila* | LP107_2017_92 | 6,86 | 0,39 | | | 0,66 | 0,58 | 10,80 | | 2,01 | | | 1,35 | 0,61 | | | 2,54 | | 36,04 | | 10,96 | | 9,84 | | 0,00 | | 0,25 | | 4,68 | | 6,25 | | 1,60 | | 0,29 | | 1,98 | 0,71 | 1,21 |
| *L. pneumophila* | LP108_2017_94 | 4,44 | 0,36 | | | 1,36 | 0,45 | 11,63 | | 1,65 | | | 1,78 | 0,67 | | | 2,27 | | 25,59 | | 17,34 | | 17,69 | | 0,00 | | 0,35 | | 5,69 | | 3,15 | | 1,15 | | 0,00 | | 2,14 | 0,43 | 0,67 |
| *L. pneumophila* | LP109_2017_96 | 5,67 | 0,37 | | | 0,71 | 0,50 | 10,59 | | 2,07 | | | 1,56 | 0,56 | | | 2,28 | | 32,27 | | 10,85 | | 11,71 | | 0,00 | | 0,33 | | 5,27 | | 6,29 | | 2,13 | | 0,37 | | 2,77 | 0,94 | 1,96 |
| *L. pneumophila* | LP110_2017_100 | 6,06 | 0,00 | | | 0,86 | 0,53 | 10,57 | | 1,81 | | | 1,28 | 0,76 | | | 2,50 | | 29,40 | | 12,38 | | 12,05 | | 0,00 | | 0,20 | | 5,00 | | 6,55 | | 1,51 | | 0,36 | | 2,73 | 0,59 | 1,81 |
| *L. pneumophila* | LP111_2017_2915_A1 | 8,01 | 0,00 | | | 0,69 | 0,63 | 11,48 | | 2,03 | | | 1,01 | 0,50 | | | 3,14 | | 34,36 | | 12,94 | | 9,20 | | 0,00 | | 0,39 | | 4,72 | | 5,08 | | 1,38 | | 0,31 | | 1,98 | 0,54 | 1,18 |
| *L. pneumophila* | LP112_2017_2921_A1 | 6,70 | 0,00 | | | 0,66 | 0,56 | 9,97 | | 1,56 | | | 1,06 | 0,55 | | | 2,51 | | 32,40 | | 11,42 | | 12,01 | | 0,00 | | 0,43 | | 5,34 | | 4,30 | | 1,65 | | 0,42 | | 3,44 | 1,05 | 3,33 |
| *L. pneumophila* | LP113_2017_3830 | 5,70 | 0,29 | | | 0,79 | 0,51 | 9,49 | | 1,68 | | | 1,61 | 0,59 | | | 0,84 | | 35,64 | | 13,98 | | 14,17 | | 0,00 | | 0,26 | | 5,28 | | 1,75 | | 1,39 | | 0,39 | | 2,66 | 0,81 | 1,82 |
| *L. pneumophila* | LP114_2017_4027 | 8,91 | 0,56 | | | 1,00 | 0,62 | 12,98 | | 3,29 | | | 1,78 | 1,00 | | | 2,69 | | 29,80 | | 14,18 | | 9,10 | | 0,00 | | 0,30 | | 3,60 | | 5,31 | | 1,20 | | 0,22 | | 1,24 | 0,54 | 0,59 |
| *L. pneumophila* | LP116_2017_10304 | 3,84 | 0,32 | | | 1,15 | 0,37 | 10,07 | | 1,83 | | | 1,65 | 0,37 | | | 1,70 | | 27,59 | | 16,72 | | 18,34 | | 0,31 | | 0,23 | | 5,20 | | 2,64 | | 0,89 | | 0,22 | | 3,07 | 0,67 | 2,74 |
| *L. pneumophila* | LP118_2017_13076 | 6,28 | 0,00 | | | 0,49 | 0,45 | 10,03 | | 1,94 | | | 0,97 | 0,63 | | | 3,19 | | 32,67 | | 9,51 | | 8,40 | | 0,00 | | 0,31 | | 5,17 | | 6,56 | | 1,88 | | 0,49 | | 3,33 | 1,18 | 3,23 |
| *L. pneumophila* | LP119_2017_13083 | 6,31 | 0,00 | | | 0,59 | 0,49 | 9,88 | | 1,69 | | | 1,05 | 0,62 | | | 3,03 | | 33,22 | | 10,30 | | 9,83 | | 0,00 | | 0,26 | | 5,20 | | 5,29 | | 1,83 | | 0,51 | | 3,24 | 1,26 | 3,86 |
| *L. pneumophila* | LP120_2017_13904 | 5,94 | 0,41 | | | 0,67 | 0,46 | 11,03 | | 2,84 | | | 1,58 | 0,44 | | | 2,06 | | 35,80 | | 14,05 | | 10,05 | | 0,22 | | 0,19 | | 4,10 | | 5,24 | | 1,11 | | 0,18 | | 1,52 | 0,54 | 1,57 |
| *L. pneumophila* | LP121_2017_13917 | 5,92 | 0,00 | | | 0,62 | 0,50 | 10,27 | | 1,97 | | | 1,39 | 0,50 | | | 2,19 | | 34,57 | | 11,27 | | 10,81 | | 0,00 | | 0,34 | | 5,72 | | 5,30 | | 1,81 | | 0,41 | | 2,79 | 0,98 | 2,04 |
| *L. pneumophila* | LP122_2017_14194 | 3,62 | 0,40 | | | 1,18 | 0,67 | 9,44 | | 1,58 | | | 2,04 | 0,53 | | | 0,95 | | 25,37 | | 14,75 | | 19,27 | | 0,00 | | 0,36 | | 5,81 | | 2,79 | | 2,62 | | 0,40 | | 3,42 | 0,80 | 2,20 |
| *L. pneumophila* | LP123_2017_14198 | 5,01 | 0,00 | | | 0,64 | 0,64 | 9,26 | | 1,19 | | | 1,05 | 0,77 | | | 2,56 | | 29,78 | | 11,75 | | 12,85 | | 0,00 | | 0,40 | | 5,92 | | 4,50 | | 1,86 | | 0,52 | | 4,08 | 1,17 | 3,91 |
| *L. pneumophila* | LP124_2017_14204 | 8,13 | 0,00 | | | 1,27 | 0,62 | 11,36 | | 2,97 | | | 1,54 | 1,36 | | | 3,10 | | 26,83 | | 16,72 | | 11,98 | | 0,00 | | 0,00 | | 3,60 | | 4,28 | | 1,32 | | 0,00 | | 1,76 | 0,40 | 0,90 |
| *L. pneumophila* | LP125_2017_14205 | 4,68 | 0,00 | | | 0,71 | 0,38 | 8,92 | | 2,00 | | | 1,25 | 0,76 | | | 1,91 | | 31,60 | | 13,50 | | 13,73 | | 0,00 | | 0,00 | | 5,50 | | 2,83 | | 1,93 | | 0,39 | | 4,05 | 1,48 | 4,21 |
| *L. pneumophila* | LP126_2017_14977 | 8,50 | 0,74 | | | 1,59 | 0,70 | 13,55 | | 3,58 | | | 2,16 | 1,11 | | | 1,59 | | 25,65 | | 17,16 | | 11,11 | | 0,00 | | 0,00 | | 3,18 | | 3,92 | | 1,51 | | 0,00 | | 1,24 | 0,57 | 0,82 |
| *L. pneumophila* | LP127_2017_14984 | 3,49 | 0,25 | | | 0,92 | 0,34 | 7,27 | | 1,56 | | | 1,88 | 0,37 | | | 0,81 | | 29,11 | | 18,26 | | 19,93 | | 0,10 | | 0,29 | | 5,15 | | 0,80 | | 1,88 | | 0,33 | | 3,51 | 1,02 | 2,67 |
| *L. pneumophila* | LP128_2017_15006 | 4,47 | 0,39 | | | 0,78 | 0,38 | 8,07 | | 2,08 | | | 1,56 | 0,26 | | | 1,64 | | 32,80 | | 18,45 | | 14,61 | | 0,15 | | 0,27 | | 4,66 | | 1,11 | | 1,40 | | 0,25 | | 2,57 | 1,01 | 2,99 |
| *L. pneumophila* | LP129_2017_15640 | 8,19 | 0,51 | | | 0,84 | 0,57 | 13,56 | | 3,20 | | | 1,54 | 0,99 | | | 2,29 | | 33,06 | | 14,95 | | 8,38 | | 0,00 | | 0,32 | | 4,30 | | 3,66 | | 1,11 | | 0,21 | | 1,20 | 0,42 | 0,54 |
| *L. pneumophila* | LP130_2017_15642 | 6,24 | 0,00 | | | 0,64 | 0,45 | 11,52 | | 2,50 | | | 1,63 | 0,67 | | | 1,75 | | 37,23 | | 12,25 | | 9,90 | | 0,00 | | 0,25 | | 5,90 | | 2,98 | | 1,46 | | 0,34 | | 2,17 | 0,76 | 1,36 |
| *L. pneumophila* | LP132_2017_15856 | 2,77 | 0,19 | | | 1,33 | 0,33 | 6,58 | | 1,16 | | | 2,21 | 0,33 | | | 0,49 | | 26,34 | | 15,79 | | 26,91 | | 0,00 | | 0,34 | | 5,01 | | 0,93 | | 1,68 | | 0,30 | | 4,14 | 0,90 | 2,21 |
| *L. pneumophila* | LP133_2017_15858 | 3,09 | 0,24 | | | 1,00 | 0,37 | 8,33 | | 1,26 | | | 1,71 | 0,39 | | | 0,96 | | 28,82 | | 15,01 | | 19,80 | | 0,17 | | 0,40 | | 6,50 | | 1,92 | | 1,67 | | 0,33 | | 3,87 | 1,04 | 3,07 |
| *L. pneumophila* | LP134_2017_16867 | 6,90 | 0,00 | | | 0,73 | 0,48 | 10,32 | | 1,77 | | | 1,85 | 0,88 | | | 2,68 | | 31,78 | | 10,19 | | 11,20 | | 0,00 | | 0,00 | | 4,91 | | 6,83 | | 1,64 | | 0,43 | | 2,78 | 0,71 | 1,69 |
| *L. pneumophila* | LP137_2017_17239 | 9,48 | 0,45 | | | 0,72 | 0,77 | 12,33 | | 3,29 | | | 1,60 | 1,44 | | | 2,22 | | 36,81 | | 11,48 | | 7,20 | | 0,00 | | 0,30 | | 3,92 | | 3,26 | | 1,09 | | 0,29 | | 1,14 | 0,67 | 0,60 |
| *L. pneumophila* | LP138_2017_17845 | 7,76 | 0,68 | | | 0,72 | 0,54 | 13,67 | | 4,22 | | | 1,38 | 0,50 | | | 0,33 | | 35,36 | | 14,80 | | 6,31 | | 0,00 | | 0,18 | | 4,01 | | 5,70 | | 1,04 | | 0,16 | | 1,02 | 0,56 | 0,96 |
| *L. pneumophila* | LP140_2017_19555 | 6,17 | 0,00 | | | 0,58 | 0,40 | 11,05 | | 2,22 | | | 1,43 | 0,73 | | | 1,90 | | 37,22 | | 10,92 | | 9,49 | | 0,00 | | 0,30 | | 5,63 | | 4,58 | | 1,74 | | 0,35 | | 2,30 | 0,92 | 1,92 |
| *L. pneumophila* | LP141_2017_19559 | 7,24 | 0,45 | | | 0,72 | 0,50 | 12,55 | | 2,77 | | | 1,54 | 0,78 | | | 2,31 | | 35,22 | | 13,11 | | 8,91 | | 0,00 | | 0,26 | | 4,97 | | 3,73 | | 1,17 | | 0,27 | | 1,49 | 0,56 | 0,79 |
| *L. pneumophila* | LP144_2017_20670 | 7,77 | 0,38 | | | 0,42 | 0,33 | 14,24 | | 3,58 | | | 1,16 | 0,84 | | | 3,01 | | 39,36 | | 15,07 | | 6,42 | | 0,23 | | 0,07 | | 4,24 | | 0,58 | | 0,53 | | 0,00 | | 0,78 | 0,36 | 0,62 |
| *L. pneumophila* | DSM 7513 | 6,50 | 0,30 | | | 0,66 | 0,62 | 9,35 | | 1,72 | | | 1,47 | 0,57 | | | 1,22 | | 43,13 | | 10,76 | | 10,33 | | 0,00 | | 0,25 | | 5,14 | | 2,50 | | 1,18 | | 0,37 | | 2,01 | 0,58 | 1,18 |
| *L. pneumophila* | DSM 25071 | 6,01 | 0,31 | | | 1,09 | 0,49 | 9,95 | | 2,41 | | | 2,54 | 0,80 | | | 1,05 | | 34,42 | | 13,13 | | 13,38 | | 0,11 | | 0,29 | | 4,76 | | 2,06 | | 1,78 | | 0,35 | | 2,31 | 0,94 | 1,61 |
| *L. pneumophila* | DSM 25182 | 6,17 | 0,48 | | | 0,55 | 0,61 | 12,14 | | 2,43 | | | 1,15 | 0,43 | | | 2,59 | | 33,49 | | 13,01 | | 8,34 | | 0,36 | | 0,28 | | 6,09 | | 5,09 | | 1,54 | | 0,29 | | 2,14 | 0,86 | 1,97 |
| *L. pneumophila* | DSM 25184 | 7,83 | 0,40 | | | 0,60 | 0,85 | 13,34 | | 2,58 | | | 1,33 | 0,62 | | | 3,12 | | 36,34 | | 11,26 | | 7,07 | | 0,37 | | 0,41 | | 5,06 | | 4,16 | | 1,33 | | 0,26 | | 1,54 | 0,63 | 0,89 |
| *L. longbeachae* | DSM 10572 | 6,19 | 0,34 | | | 0,62 | 0,31 | 13,14 | | 2,51 | | | 1,85 | 0,00 | | | 0,61 | | 21,20 | | 27,45 | | 14,19 | | 0,00 | | 0,35 | | 4,68 | | 2,46 | | 1,48 | | 0,19 | | 1,01 | 0,18 | 0,17 |
| *L. micdadei* | DSM 16640 | 0,85 | 0,14 | | | 0,59 | 0,51 | 39,19 | | 1,68 | | | 2,49 | 0,29 | | | 1,26 | | 13,47 | | 5,97 | | 5,35 | | 3,22 | | 0,26 | | 20,29 | | 2,17 | | 0,75 | | 0,14 | | 0,17 | 0,10 | 0,60 |
| *L. bozemanii* | DSM 16523 | 2,77 | 0,12 | | | 0,42 | 0,19 | 29,50 | | 1,92 | | | 3,79 | 0,00 | | | 0,17 | | 11,80 | | 9,59 | | 13,10 | | 0,22 | | 0,63 | | 8,71 | | 5,58 | | 6,85 | | 0,48 | | 1,32 | 1,12 | 0,48 |
| *L. dumoffii* | DSM 17625 | 2,25 | 0,17 | | | 0,38 | 0,25 | 37,71 | | 0,27 | | | 0,44 | 0,00 | | | 0,53 | | 15,46 | | 7,54 | | 8,45 | | 0,00 | | 1,08 | | 14,47 | | 8,43 | | 0,88 | | 0,18 | | 0,73 | 0,00 | 0,18 |
| *L. gormanii* | DSM 25296 | 4,54 | 0,16 | | | 0,42 | 0,23 | 21,93 | | 2,72 | | | 4,41 | 0,00 | | | 0,18 | | 18,94 | | 9,46 | | 9,27 | | 0,00 | | 0,49 | | 9,17 | | 7,17 | | 5,62 | | 0,39 | | 0,98 | 1,53 | 0,60 |
